# Taxonomy Identification and Phytotoxic Activities of Pectolytic Bacteria Isolated from Diseased Plants of Phalaenopsis Blume (Orchidaceae)

**DOI:** 10.3390/plants15121901

**Published:** 2026-06-18

**Authors:** Anastasiya A. Bychkova, Xenia D. Desneva, Milana M. Filippova, Maksim N. Sokolov, Denis Y. Kushpetiuk, Natalia A. Makeeva, Julia A. Balabanova, Gennady L. Burygin, Yuliya V. Zaitseva

**Affiliations:** 1 Department of Botany and Microbiology, Faculty of Biology and Ecology, P.G. Demidov Yaroslavl State University, 150003 Yaroslavl, Russia; xenia.des@mail.ru (X.D.D.); melsudbi@yandex.ru (M.N.S.); zjv9@mail.ru (Y.V.Z.); 2 Department of Plant Breeding, Selection, and Genetics, Institute of Genetics and Agronomy, Vavilov University, 410012 Saratov, Russia; deniskushp@gmail.com (D.Y.K.); nmakeeva15a@gmail.com (N.A.M.); yulia-kusmarceva@yandex.ru (J.A.B.); 3 Institute of Biochemistry and Physiology of Plants and Microorganisms, Saratov Scientific Centre of the Russian Academy of Sciences, 13 Prospekt Entuziastov, 410049 Saratov, Russia; 4 Department of Organic and Bioorganic Chemistry, Institute of Chemistry, Saratov State University, 83 Astrakhanskaya Street, 410012 Saratov, Russia

**Keywords:** *Phalaenopsis*, soft rot, pectolytic activity, biofilm, swimming, swarming, potential phytotoxicity

## Abstract

Orchid plants, due to their high aesthetic qualities of large inflorescences, long flowering period, and ease of care, have high commercial potential; however, when grown industrially in factories, they are susceptible to infectious diseases. In this study, we isolated from *Phalaenopsis* spp. plants epiphytic, rhizospheric, and endophytic bacteria associated with soft rot symptoms. Twenty-nine isolates exhibiting pectolytic activity were identified as strains of the genera *Bacillus*, *Klebsiella*, *Microbacterium*, *Paenibacillus*, *Paracidovorax*, *Pseudomonas*, and *Psychrobacillus* based on 16S rRNA analysis. These isolates were tested for their ability to produce cellulase, amylase, sucrase, proteinase, and lipase; to form biofilms; and to exhibit motility (swimming and swarming). Potato microplants under in vitro conditions were used as a model object for initial screening of the strains’ potential phytotoxicity. Most strains were shown to inhibit plant growth, particularly root development. Injection of suspensions of these strains into orchid leaves caused symptoms of soft rot. Thus, we isolated Gram-positive bacteria for the first time from orchid tissues with soft rot symptoms and demonstrated an association of these strains with plant tissue maceration in potato and orchids. Gram-positive bacteria with pectolytic activity are not typical pathogens of orchid soft rot and may require changes in approaches to the monitoring of phytopathogens for this group of plants.

## 1. Introduction

Orchidaceae is the largest family of flowering plants, comprising approximately 736 genera and over 25,000 species [1,2]. Orchids are widespread and inhabit various climatic zones, including extreme desert and highland conditions; however, the greatest diversity of plants in this family is found in tropical countries [3]. Orchids have high potential for commercialization, as they are widely used as houseplants, grown for cut flowers, and used for the extraction of medicinal compounds [4]. The leading orchid producers are countries such as Australia, the Netherlands, New Zealand, Singapore, Thailand, and Japan [4,5,6]. The most popular are representatives of the genus *Phalaenopsis* Blume, accounting for over 75% of the entire orchid market [7]. Due to their high aesthetic qualities, long flowering period, and ease of care, plants of this genus are in demand among consumers [8]. In Russia, industrial production of *Phalaenopsis* is just beginning to develop. Orchid-growing enterprises operate in the Yaroslavl (Gorkunov Group), Tomsk (Siberian Orchids LLC), and Moscow (Baikal Flowers LLC) regions.

Growing orchids on an industrial scale is complicated by the long development cycle of the plants, special requirements for the substrate and cultivation conditions, and susceptibility to infections. One of the most dangerous diseases, leading to significant losses in orchid production, is bacterial soft rot, caused by representatives of several genera of Gram-negative bacteria: *Pectobacterium*, *Klebsiella*, *Serratia*, *Enterobacter*, *Citrobacter*, *Providencia*, and *Pseudomonas* [9]. Symptoms of soft rot include the appearance of light-green, water-soaked spots on the leaves, which gradually increase in size and acquire a brown tint [10,11].

Orchid cultivation conditions, such as high humidity and temperature, can promote the rapid spread of infection. Particularly critical is impaired air exchange, which can lead to water accumulation in the leaf axils [12]. Under such conditions, a latent infection transitions to an active form of the disease, accompanied by the appearance of characteristic soft rot symptoms. Soft rot pathogens spread rapidly in greenhouses, causing widespread plant damage. In commercial orchid production, yield losses due to infection can reach 30% or more [9]. Therefore, studying the causative agents of soft rot and developing methods for their prevention is an urgent task. There are no effective treatments for this disease, so preventive measures are very important, including early detection and identification of pathogens in planting material. Therefore, isolating orchid phytopathogens and studying their phylogenetic diversity is an important task in agricultural microbiology.

The aim of this study was to isolate, identify, and evaluate the phytotoxicity of pectolytic microorganisms that cause soft rot symptoms in *Phalaenopsis* Blume (Orchidaceae) under greenhouse conditions.

## 2. Materials and Methods

### 2.1. Plant Material and Isolation of Bacterial Strains

Three-year-old generative plants (with 3–4 leaves) with signs of soft rot on the leaves were selected for the study. Plant material was collected under aseptic conditions at the “Yaroslavsky” greenhouse complex (Dubki, Yaroslavl region, Russia). Plants were grown at a temperature of 25 °C and a humidity of 60–80%. Twenty-one plants from seven different batches were examined (Table S1). Preliminary testing of these plants was performed using the commercial real-time PCR test system *Pectobacterium* spp.-PB (Synthol, Moscow, Russia), according to the manufacturer’s instructions. The analysis was carried out by employees of the “Yaroslavsky” greenhouse complex during the purchase of primary planting material.

A 5 g sample of leaves, roots, and substrate was collected for microbiological analysis. Epiphytic strains were isolated by rinsing the leaves, which were previously rinsed with 50 mL of sterile tap water. Rhizosphere strains were isolated from roots and substrate fragments, which were collected and placed in a flask with 50 mL of sterile tap water and incubated for 1 h at 28 °C and 150 rpm. To isolate endophytic strains, the plant material was washed with 50 mL of sterile water for 10 min, then soaked in 70% ethanol for 2 min, and then washed in 10% sodium hypochlorite for 20 min. After washing three times in sterile water, the material was homogenized. A series of tenfold dilutions was prepared from the resulting leaf and root washes and homogenates, from which 50 μL of dilutions were inoculated into Petri dishes with LB agar medium (tryptone, 10 g/L; yeast extract, 5 g/L; NaCl, 5 g/L; agar, 15 g/L). The cultures were incubated in an incubator at 28 °C for 3 days. The resulting isolated colonies were plated on LB agar medium. The strains were stored on a semi-liquid LB medium (agar, 5 g/L) under mineral oil at a temperature of +4 °C.

### 2.2. Determination of Pectolytic Activity

To select strains with pectolytic activity, we assessed their growth ability on MP-7 pectate medium (HiMedia Laboratories, Mumbai, India) and their ability to macerate potato tuber tissue [13]. Potato tubers were washed with soapy water and then soaked in a 10% sodium hypochlorite solution for 20 min. Aseptically cut potato cubes were placed in Petri dishes on damp filter paper, inoculated with the isolated strains, and incubated for 3 days at 28 °C. Signs of maceration were considered to indicate pectate lyase activity.

### 2.3. Determination of Enzymatic Activities

The activities of hydrolytic and antioxidant enzymes were determined for bacterial isolates that caused plant tissue maceration.

Proteolytic activity was assessed on a solid LB medium supplemented with sterile skim milk (330 mL/L) by the presence of a clearing zone on the medium [14]. The ability to synthesize sucrase was studied on a Giss medium with sucrose and the indicator VR [15]. Lipase activity was assessed on a solid LB medium with 1 mM CaCl_2_ and 1% Tween-20. The presence of enzymatic activity in the strain was determined visually by the formation of clusters of insoluble calcium compounds around the colony [14]. Amylolytic activity was assessed on a solid LB medium supplemented with starch (15 g/L). The results were visualized using an aqueous iodine solution [16]. The absence of bluing of the medium was recorded as the presence of amylase enzyme activity. Cellulase activity was determined on a medium with carboxycellulose: K_2_HPO_4_, 1.0 g/L; KH_2_PO_4_, 1.0 g/L; NH_4_Cl, 2.5 g/L; CaCl_2_, 0.1 g/L; NaCl, 0.1 g/L; MgCl_2_, 0.5 g/L; carboxymethylcellulose, 10 g/L; yeast extract, 1.0 g/L; agar, 20 g/L [17]. The results were visualized using a 0.1% aqueous solution of Congo red, followed by washing the cultures with 1 M NaCl [18]. The appearance of a clearing zone in the medium around the colony of the strain was considered a positive result. All hydrolytic enzymatic activities were assessed after 48 h of cultivation at a temperature of 28 °C.

The activity of amylase, cellulase, and protease enzymes was estimated by the diameter of the clearing zone around the colony. The enzyme activity index (EI) was calculated using the following formula:


EI = (colony diameter + clear zone)/colony diameter.

Oxidase and catalase activities were assessed according to the standard method using a 1% solution of *N*,*N*-dimethyl-*p*-phenylenediamine sulfate and 3% hydrogen peroxide, respectively [19,20].

### 2.4. Evaluation of the Ability of Bacteria to Form Biofilms

The overnight bacterial culture was added to liquid LB medium at a ratio of 1:100 (to a concentration of approximately 10^7^ CFU/mL) and grown in a 96-well plate for 24 h at 28 °C at 150 rpm. The LB liquid medium was used as a negative control, *Pseudomonas aeruginosa* PAO1 was used as a positive control. Planktonic cell growth was then determined by measuring the optical density of the bacterial culture at 620 nm (OD_620_). The culture was then decanted, and the wells of the plate were washed three times with sterile water to remove planktonic cells. The biofilms were then stained with 0.1% crystal violet solution for 45 min, the wells were washed five times with sterile water, and the dye was extracted from the biofilms with 96% ethanol. The level of biofilm formation was assessed by measuring the optical density of the solution at 595 nm (OD_595_) [21].

The ability to form biofilm was considered positive at a cutoff optical density of 0.08, which was arbitrarily chosen as the mean value of the negative control (nutrient medium, 0.065) plus three standard deviations of the control (0.005). The levels of biofilm formation were classified as follows. Microorganisms were considered not to form biofilm if the optical density was less than 0.08. Weak biofilm-forming microorganisms had optical density values ranging from 0.08 to 0.12. Moderate biofilm-forming microorganisms had optical density values ranging from 0.12 to 0.54, and strong biofilm-forming microorganisms had optical density values above 0.54 [22].

### 2.5. Determination of Bacterial Swarming and Swimming Abilities

An overnight bacterial culture was added to liquid LB medium at a ratio of 1:100 and incubated for 2–3 h at 28 °C at 150 rpm until the culture reached the exponential growth phase. For determination of bacterial swarming ability, two microliters of the culture were then applied to the surface of a semi-solid M9 medium (Na_2_HPO_4_, 6.0 g/L; KH_2_PO_4_, 3.0 g/L; NaCl, 0.5 g/L; NH_4_Cl, 1.0 g/L; agar, 6.0 g/L) supplemented with 1 mL of 0.1 M CaCl_2_, 1 mL of 1 M MgSO_4_, 4 g/L glucose, and 5 g/L casamino acid and incubated for 48 h at 28 °C. Determination of bacterial swimming ability was performed similarly except for the addition of agar, 3.0 g/L. The presence of cell migration zones on the surface was determined visually [23].

Based on individual measurements of the colony’s growth zone on the medium, the average area for each strain was calculated to determine motility characteristics. If the area value was greater than 40 mm^2^, the bacterium was considered motile. *Pseudomonas aeruginosa* PAO1 was considered a positive control [22].

### 2.6. Taxonomic Identification

DNA extraction was performed from pure bacterial cultures grown in liquid LB medium for 24 h at 28 °C using the ExtractDNA kit (Eurogen, Moscow, Russia) according to the manufacturer’s instructions. Taxonomic identification of the pectolytic strains was performed based on the 16S rRNA gene sequence using universal primers 356F (5′-ACWCCTACGGGWGGCWGC-3′) and 1064R (5′-AYCTCACGRCACGAGCTGAC-3′) and the HS-Taq PCR-Color (2x) BioMaster kit (Biolabmix, Moscow, Russia). Bacteria were identified using the GenBank database (BLASTn, rRNA/ITS databases) based on primary nucleotide sequence analysis. Phylogenetic trees were constructed using multiple nucleotide sequence alignments. The alignment was performed using MEGA12 software (version 12.0.10) [24] with the CLUSTALW algorithm. The Neighbor-Joining method and the Tajima-Nei model were used to construct the tree.

### 2.7. Effect of Bacterial Inoculation on Potato Microplants

The ability of the isolated bacterial strains to inhibit plant growth and cause disease symptoms was assessed in vitro using the model object, potato (*Solanum tuberosum* L. cultivar Nevsky) microplants from the plant collection maintained by the Department of Plant Breeding, Selection, and Genetics of the Institute of Genetics and Agronomy at Vavilov University (Saratov, Russia). Microplants were grown as described by Kargapolova et al. [25] with modifications. Potato microcuttings, each containing one node, were placed in test tubes fitted with cotton-gauze plugs containing 5 mL of a hormone-free liquid Murashige–Skoog nutrient medium [26]. Microplants were grown under the following conditions: temperature, 24 °C; humidity, 60%; light intensity, 60 µM/(m^2^ s); day length, 16 h. Then, 10-day-old microplants were inoculated with 50 μL portions of a bacterial suspension (10^8^ cells/mL) in 5 mL of the nutrient medium. The inoculated microplants were grown for 20 days. The controls were uninoculated plants grown on a sterile nutrient medium. Morphological characteristics such as shoot length, maximum root length, shoot fresh weight, and root fresh weight were recorded for 30-day-old microplants.

Each strain was tested in three independent experiments, each using 10 microplants. The fresh weight of the shoot and roots of each microplant was measured three times (technical replicates). Before the inferential analyses, data were examined for normality (Shapiro–Wilk test) and homogeneity of variances (Bartlett’s test) to ensure the validity of subsequent procedures. Mean values of plant morphometric data from three experiments were processed by one-way ANOVA (*p* ≤ 0.05). Honest significant differences (HSD_0.05_) were determined at a significance level of 95% (*p*  =  0.05). Values followed by different letters differed significantly at *p*  ≤  0.05 according to Tukey’s multiple range test using Statistica 12 software (StatSoft, Inc., Palo Alto, CA, USA). Measured data are presented as the mean  ±  confidence interval for a 95% significance level.

The severity of the disease and the morbidity index (DI) were assessed according to the following assessment criteria (damage levels): 0, no symptoms; 1, slight damage, less than 5% of the whole plant area; 3, damaged areas make up 6–10% of the whole plant area; 5, the degree of damage is 11–20% of the whole plant area; 7, damage up to 40% of the whole plant area; 9, damaged areas occupy more than 40% of the whole area [27]. The DI value for each of the variants was calculated using the following formula:

DI = [∑(NDL × GLDS)/(TNIL × 9)] × 100

where NDL is the number of diseased leaves at each level; GLDS is the grade level of disease severity; TNIL is the total number of investigated leaves; and 9 is the highest grade level.

### 2.8. Effect of Bacterial Inoculation on Phalaenopsis in Planta

The ability of the isolated bacterial strains to infect plants and cause disease symptoms was assessed in planta using 3-year-old plants of *Phalaenopsis* spp. (Orchidaceae). The strain cultures were grown on LB medium for 24 h and then centrifuged at 13,000 rpm for 5 min. The supernatant was removed, and the pellet was resuspended in sterile water. Next, 10 μL of bacterial cell suspension (1 × 10^6^ CFU/mL) was injected into the parenchyma of *Phalaenopsis* leaves using a syringe. An injection of 10 μL of sterile water was used as a control. After 5 days, the appearance of plant tissue damage symptoms at the injection site was visually analyzed.

## 3. Results

### 3.1. Isolation of Bacterial Strains and Evaluation of Their Pectolytic Activity

**Figure 1:**
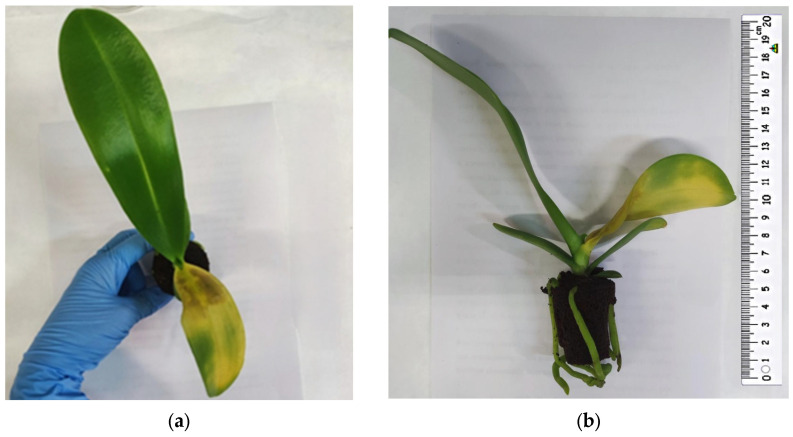
*Phalaenopsis* spp. plants showing signs of bacterial soft rot: top view (**a**) and side view (**b**).

During this study, vegetative organs of *Phalaenopsis* orchids with visible signs of bacterial soft rot were collected (Figure 1). Preliminary testing of these plants with the commercial real-time PCR test system *Pectobacterium* spp.-PB did not reveal the presence of the pathogen in the collected samples. A total of 180 bacterial isolates were isolated from *Phalaenopsis* spp. tissues, including 20 isolates from the phyllosphere, 127 isolates from the endosphere, and 33 isolates from the rhizosphere.

Proteolytic enzymes are among the main factors responsible for plant pathogenicity. In the first stage, isolates were tested for pectolytic activity. Twenty-nine strains exhibited vigorous growth on MP-7 pectate medium and caused maceration of plant tissue (droplets of turbid liquid with a specific odor). We described these strains as pectolytic, and further experiments were conducted exclusively with them.

### 3.2. Taxonomic Identification of Isolated Pectolytic Bacteria

**Figure 2:**
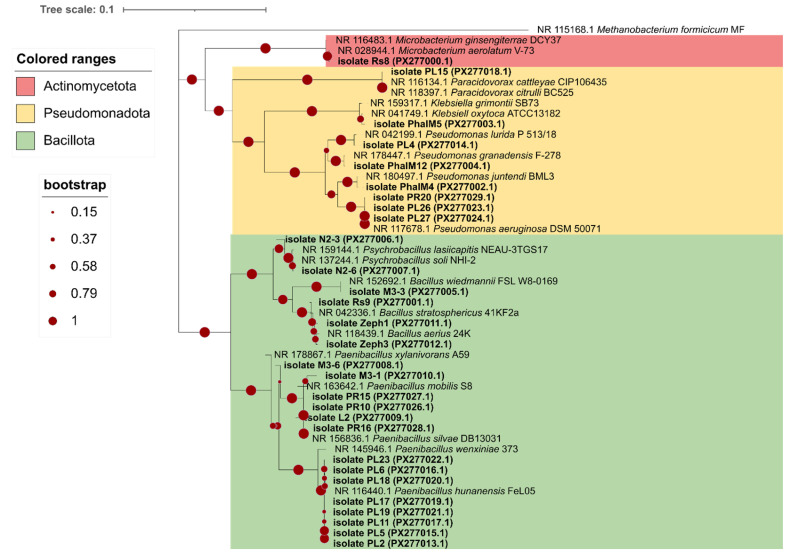
Phylogenetic tree of isolated pectolytic bacterial strains based on 16S rRNA gene nucleotide sequences using the Neighbor-Joining method. The nucleotide sequence of *Methanobacterium formicicum* MF was used as the root.

Identification of the selected bacterial strains with pectolytic activity by the 16S rRNA gene sequence allowed them to be assigned to 3 phyla (Actinomycetota, Bacillota, and Pseudomonadota) and 7 genera (*Bacillus*, *Paenibacillus*, *Klebsiella*, *Microbacterium*, *Paracidovorax*, *Pseudomonas*, and *Psychrobacillus*) (Figure 2; Table S2).

Epiphytic strains are represented by the genera *Pseudomonas* (2 isolates), *Klebsiella* (1 isolate), and *Bacillus* (2 isolates); endophytes belong to the genera *Psychrobacillus* (2 isolates), *Paenibacillus* (11 isolates), *Pseudomonas* (3 isolates), *Bacillus* (1 isolate), and *Paracidovorax* (1 isolate); root endosphere strains are represented by *Microbacterium* (1 isolate), *Bacillus* (1 isolate), *Pseudomonas* (1 isolate), and *Paenibacillus* (3 isolates).

### 3.3. Enzymatic Activity of Pectolytic Strains

The presence of plant cell wall degrading enzymes (PCWDEs) and antioxidant defense enzymes plays an important role in phytopathogenesis, facilitating the penetration of the microorganism into the host organism and providing protection against reactive oxygen species produced by plants. The activities of several hydrolytic and antioxidant enzymes were determined for the 29 selected strains. The results are presented in Table 1.

Strains *Pseudomonas* sp. PhalM12, PhalM4, PL4, PL26, PL27, and PR20 demonstrated catalase and oxidase activity. Isolates PL26 and PL27 exhibited cellulase, amylase, and sucrase activity. *Bacillus* sp. Rs9, M3-3, Zeph1, and Zeph3 possessed catalase and oxidase activity and demonstrated sucrase and cellulase activity. Protease activity was shown only for strain M3-3. Bacterial isolates L2, M3-6, M3-1, PL2, PL5, PL6, PL11, PL17, PL18, PL19, PL23, PR10, PR15, and PR16, belonging to the genus *Paenibacillus*, demonstrated a pronounced ability to utilize sucrose and cellulose and had lipase activity. Moreover, the most pronounced activity of PCWDEs was demonstrated by the strains *Bacillus* sp. Rs9, *Bacillus* sp. Zeph1, *Bacillus* sp. Zeph3, *Paenibacillus* sp. PL2, *Paenibacillus* sp. PL6, *Paenibacillus* sp. PL11, *Paenibacillus* sp. PL17, *Paenibacillus* sp. PL18, *Paenibacillus* sp. PL19, *Paenibacillus* sp. PL23, *Paenibacillus* sp. PR10, and *Paenibacillus* sp. PR15. The enzymatic activity index of these bacteria was more than 2.8.

Strains *Psychrobacillus* sp. N2-3 and N2-6 possessed cellulase and amylase activity, the ability to utilize sucrose, and demonstrated a positive reaction for catalase. Strain *Microbacterium* sp. Rs8 exhibited catalase and oxidase activity. Strain *Paracidovorax* sp. PL15 demonstrated cellulase and lipase activity. *Klebsiella* sp. PhalM5 demonstrated the presence of cellulase, protease, oxidase, and catalase.

The strains *Bacillus* sp. M3-3, *Paenibacillus* sp. M3-1, *Paenibacillus* sp. PL2, *Paenibacillus* sp. PL11, *Paenibacillus* sp. PL19, and *Paenibacillus* sp. PL23 possessed all types of enzymes that destroy plant cell walls, which may indicate a high potential for these strains to act as causative agents of plant diseases.

### 3.4. Biofilm Formation by Pectolytic Strains

The formation of biofilms by phytopathogenic microorganisms promotes effective colonization of the host plant and serves as a mechanism of protection against environmental factors unfavorable for bacteria and plant immunity. The selected isolates were assessed for their biofilm-forming ability (Table 2). It was shown that all strains except *Paenibacillus* sp. M3-1, *Paenibacillus* sp. M3-6, *Paenibacillus* sp. PL19, *Paenibacillus* sp. PR16, and *Pseudomonas* sp. PhalM12 were capable of forming biofilms. At the same time, the strains *Pseudomonas* sp. PhalM4, *Pseudomonas* sp. PL4, *Paenibacillus* sp. PL2, *Paracidovorax* sp. PL15, *Psychrobacillus* sp. N2-3, and *Psychrobacillus* sp. N2-6 formed moderate biofilms, and *Pseudomonas* sp. PL26, *Pseudomonas* sp. PL27, *Pseudomonas* sp. PR20, *Bacillus* sp. Rs9, *Bacillus* sp. Zeph1, and *Bacillus* sp. Zeph3 formed strong biofilms (OD > 0.54).

### 3.5. Assessing the Motility of Pectolytic Strains

One of the virulence factors that contributes to plant colonization is the ability of bacteria to exhibit flagellar motility. The motility of pectolytic strains was assessed by examining their swimming and swarming abilities. The results showed that all bacterial strains, except *Klebsiella* sp. PhalM5, demonstrated swimming activity. However, swarming was characteristic of only a few bacteria: *Bacillus* sp. Rs9, Zeph1, Zeph3, *Paenibacillus* sp. L2, PL11, PL17, PL18, PL19, and *Pseudomonas* sp. PL27, with a colony area greater than 40 mm^2^. The results are presented in Table 2.

### 3.6. Effect of Bacterial Inoculation on Potato Microplants in a Model Experiment

The probable phytotoxicity of the selected strains was assessed using *Solanum tuberosum* L. cultivar Nevsky under in vitro conditions. Inoculation had the most pronounced effect on parameters such as shoot length, root length, and root and shoot fresh weight (Table 3). A significant decrease in shoot length was observed for 14 strains. The strongest inhibition (more than 25%) was observed with inoculation with *Klebsiella* sp. PhalM5, *Pseudomonas* sp. PL26, and *Paenibacillus* sp. PL6 (52%, 31%, and 27%, respectively). Black spots also appeared on the underside of potato leaves in the experimental variants.

Analysis of potato shoot fresh weight revealed that 20 strains tested had a significant negative effect. Of these, 15 strains reduced fresh weight by more than 25%. The highest inhibition was observed with inoculation with *Klebsiella* sp. PhalM5, at 59% compared to the control.

The effect of inoculation on potato root length was mixed: 10 strains inhibited root elongation, 5 strains increased root length, and the remaining strains had no significant effect. *Paenibacillus* sp. strains PL5, PL6, PL11, and PL17 reduced root length by more than 50% compared to uninoculated microplants.

Inoculation of plants with the selected strains had the most pronounced negative impact on root fresh weight. A negative impact was observed in treatments with 25 strains. Twenty strains showed inhibition of more than 50%. Five strains (*Klebsiella* sp. PhalM5, *Paenibacillus* sp. PL2, PL5, PL11, and PL17) showed a decrease in root fresh weight of more than 90%.

Based on the obtained results, seven strains (*Klebsiella* sp. PhalM5, *Pseudomonas* sp. PL27, *Paenibacillus* sp. PL5, PL6, PL17, PL18, and PL23) were identified that had a significant negative impact on the development of all four potato microplant parameters. Inoculation with seven more strains (*Bacillus* sp. M3-3, *Paenibacillus* sp. L2, PL11, PL19, PL23, *Pseudomonas* sp. PhalM12, and PL26) significantly reduced the values of three microplant parameters. For three strains (*Microbacterium* sp. Rs8, *Paenibacillus* sp. M3-1, and *Paenibacillus* sp. M3-6), no inhibitory effects of inoculation were detected, but on the contrary, a significant increase in roots was observed; therefore, these three strains are not phytopathogenic in relation to potato.

The disease incidence index (DI) in plants inoculated with 15 strains was greater than 5 percent (Table 3). The highest DI values were demonstrated by *Klebsiella* sp. PhalM5, *Paenibacillus* sp. PL23, and *Paenibacillus* sp. PL2—77.8, 33.3, and 23.6 percent, respectively. In addition, these three strains caused symptoms of bacterial infection, manifested as spots on leaves and stems, wilting of leaf blades, and blackening and sliming of roots (Figure S1). Strains *Paenibacillus* sp. PL2 and *Paenibacillus* sp. PL23 are characterized by the activity of several PCWDEs (pectinase, cellulase, amylase, protease, and lipase), as well as swimming-type motility. Strains *Bacillus* sp. M3-3, *Paenibacillus* sp. M3-1, *Paenibacillus* sp. PL11, and *Paenibacillus* sp. PL19, demonstrating a variety of hydrolytic enzymes and flagellar motility, are also characterized by high and medium DI values in vitro. Strain *Klebsiella* sp. PhalM5 did not possess protease and lipase activities or motility but demonstrated the highest DI value. Thus, it can be concluded that the main virulence factors in the studied strains are the presence of pectinase, cellulase, and amylase activities.

### 3.7. Determination of Phytotoxicity of Strains in an Experiment with Phalaenopsis Plants

**Figure 3:**
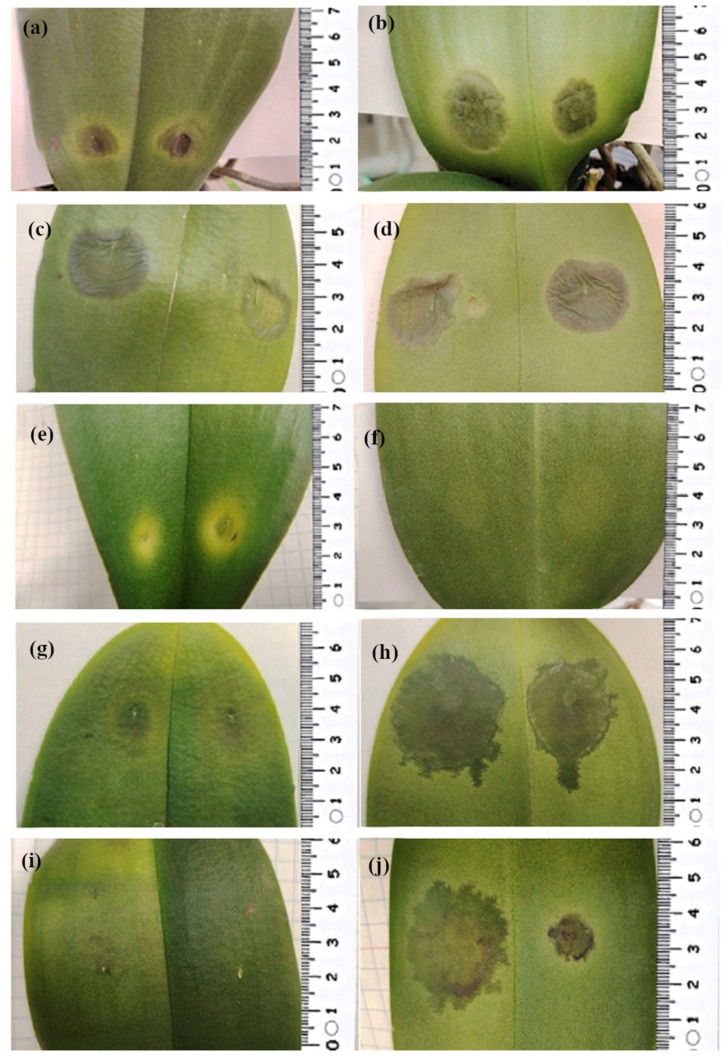
Leaves of *Phalaenopsis* spp. 5 days after with bacterial suspensions are shown: for *Microbacterium* sp. Rs8—the upper (**a**) and lower (**b**) leaf surfaces; for *Paenibacillus* sp. PL23—the upper (**c**) and lower (**d**) leaf surfaces; for *Paracidovorax* sp. PL15—the upper (**e**) and lower (**f**) leaf surfaces; for *Bacillus* sp. M3-3—the upper (**g**) and lower (**h**) leaf surfaces; for *Pseudomonas* sp. PhalM4—the upper (**i**) and lower (**j**) leaf surfaces.

An in planta phytotoxicity assay on mature *Phalaenopsis* plants revealed that 24 of the strains tested caused leaf spot formation (Figure 3 and Figure S2). The results are presented in Table 4. Bacteria from the genera *Bacillus*, *Paenibacillus*, *Microbacterium*, *Psychrobacillus*, and some representatives of the genus *Pseudomonas* caused dark, water-soaked spots ranging from 0.5 to 3 cm in diameter, or less commonly, yellow-brown spots (some *Paenibacillus*), at the sites of strain inoculation. In some cases, a distinct necrotic zone formed in the center of the spot, characterized by tissue drying and blackening (*Microbacterium* sp. RS8 and *Paenibacillus* sp. PL18). At the injection site, the strain *Klebsiella* sp. PhalM5 forms a small (up to 0.7 cm) depression in the leaf, which turns brown due to subsequent necrosis of the plant tissue; no tissue hydration is observed. *Paracidovorax* sp. PL15 also causes pigmentation disturbances at the site of cell suspension inoculation, with yellow spots appearing with pits in the center, where maceration of the orchid leaf parenchyma occurs. *Bacillus* sp. Rs9, *Paenibacillus* sp. L2, *Pseudomonas* sp. PL4, *Pseudomonas* sp. PL26, and *Pseudomonas* sp. PL27 strains did not cause changes in plant tissue. No leaf damage was observed with sterile water injection.

Bacterial strains that cause signs of leaf tissue maceration under in planta conditions are characterized by the activity of various PCWDEs, as well as swimming-type flagellar motility. The presence of amylase, protease, and lipase can vary, while the presence of pectinase and cellulase enzymes is necessary for the manifestation of phytotoxic properties.

## 4. Discussion

The natural habitat of orchids promotes the development of beneficial symbiotic interactions with various microorganisms [28]. Most of the published works on the microbiological studies of orchids are devoted to endophytic communities, since they play an important role in the reproduction and survival of these plants [29]. At the same time, the issues of pathogenic microorganisms affecting plants of this family have been poorly studied. Currently, in greenhouse farms engaged in the industrial cultivation of orchids throughout the world, the problem of the spread of soft rot pathogens is acute. The main causative agents of rot in *Phalaenopsis*, according to published data, are predominantly Gram-negative bacteria of the Pectobacteriaceae family, in particular *Pectobacterium* spp. and *Dickeya* spp. [29,30]. There is evidence that representatives of the genera *Acidovorax*, *Erwinia*, and *Pseudomonas* are also capable of causing symptoms of soft rot and bacterial brown spot of orchids [30,31,32].

In this study, bacteria with pectolytic activity were isolated from tissues of *Phalaenopsis* spp. with signs of rot. Preliminary analysis of the affected plant material using a commercial PCR test system did not reveal the presence of bacteria of the genus *Pectobacterium*. Taxonomic identification based on the 16S rRNA test showed the presence among the isolated strains of representatives of both Gram-negative bacteria of the genera *Pseudomonas*, *Klebsiella*, and *Paracidovorax*, and Gram-positive bacteria, previously not described as associated with orchid soft rot diseases, from the genera *Bacillus*, *Paenibacillus*, *Microbacterium*, and *Psychrobacillus*. Thus, it can be assumed that representatives of these genera can act as causative agents of phalaenopsis bacterial diseases in this enterprise.

The literature analysis showed that representatives of the genera *Paracidovorax* and *Pseudomonas* are considered typical causative agents of orchid bacteriosis, and bacteria of the genus *Klebsiella* were cultured from the affected organs of these plants [9]. Thus, the genus *Paracidovorax*, reclassified from the genus *Acidovorax* in 2023, is a known causative agent of bacterial spotting of a wide range of plants, including cucumbers (*Cucumis sativus* L.), watermelon (*Citrullus lanatus* (Thunb.) Matsum & Nakai), melon (*Cucumis melo* L.), corn (*Zea mays* L.), oats (*Avena sativa* L.), and wheat (*Triticum aestivum* L.) [33,34]. Orchids are also susceptible to bacterial brown leaf spot caused by *Acidovorax avenae* subsp. *cattleyae* [31,35], which indirectly confirms our findings on the involvement of *Paracidovorax* sp. PL15 in the development of *Phalaenopsis* Blume bacteriosis. Representatives of the genus *Pseudomonas* act as plant endophytes and are often used as a basis for biopreparations [36]. At the same time, some species are common phytopathogens that affect a variety of commercially significant agricultural and ornamental crops, including tomato (*Solanum lycopersicum* L.), pea (*Pisum sativum* L.), sunflower (*Helianthus mollis* Lam.), common wheat (*Triticum aestivum* L.), and cucumber (*Cucumis sativus* L.) [37]. In addition, there is evidence that bacteria of the genus *Pseudomonas* are isolated from affected tissues of orchids with signs of soft rot, which is consistent with our data [9]. Bacteria of the genus *Klebsiella* have been described primarily as an endophyte and growth promoter of plants [38,39]. However, it is reported that *the bacteria* can act as a causal agent of some infections, for example, root rot of bananas (*Musa* spp. L.), rot of onions (*Allium cepa* L.) [40], wilt and blossom-end rot of corn (*Zea mays* L.) [41].

A unique feature of this study is that it is the first report of the involvement of Gram-positive bacteria, in particular those belonging to the genera *Paenibacillus*, *Bacillus*, *Psychrobacillus* and *Microbacterium*, in the development of bacterial soft rot in plants of the Orchidaceae family. According to the literature, these bacteria typically act as endophytes of higher plants, possess a number of growth-promoting properties, and are used to protect plants from phytopathogenic microorganisms [42,43,44,45]. Furthermore, there is evidence demonstrating the involvement of representatives of these genera in phytopathogenesis. For example, there are a number of studies demonstrating the phytopathogenicity of bacteria of the genus *Paenibacillus*. Representatives of the species *Paenibacillus polymyxa* can cause storage rot of ginseng (*Panax ginseng* C.A.Mey.) and carrot (*Daucus carota* L.) [46,47], act as the causative agent of bacterial diseases of white-fleshed pitahaya (*Selenicereus undatus* (Haw.) D.R.Hunt) [48] and yellow wilt spot of three-leaved dracaena (*Dracaena trifasciata* (Prain) Mabb.) [49]. Representatives of the genus *Bacillus* can cause mango (*Mangifera indica* L.) and Asian pear (*Pyrus pyrifolia* (Burm.f.) Nak.) disease, muskmelon (*Cucumis melo* L.) and potato (*Solanum tuberosum* L.) blight, as well as ginger (*Zingiber officinale* Roscoe) root rot, bacterial spot of bean (*Phaseolus vulgaris* L.) and peach (*Prunus persica* L.), cabbage (*Brassica oleracea* L.) head rot, and bacterial rot of tomato (*Solanum lycopersicum* L.) [50,51]. In 2023, pectinolytic isolates of *Bacillus pumilus* and *Paenibacillus amyloliticus* were reported as new pathogens of potato (*Solanum tuberosum* L.) [50]. The strain *Microbacterium* sp. SUBG005 has been reported to be involved in the development of bacterial diseases of mango (*Mangifera indica* L.) and rice (*Oryza sativa* L.) [52,53]. However, no confirmed data on these genera of bacteria associated with orchid bacterial diseases have been published.

The pathogenicity of soft rot pathogens results from the secretion of extracellular plant cell wall-degrading enzymes (PCWDE). Many bacterial phytopathogens secrete a wide range of hydrolytic enzymes: pectinases, cellulases, proteases, and lipases, which facilitate the active penetration of bacteria into internal plant tissues. Among these enzymes, pectinases are considered the main exoenzymes that degrade pectin in the middle lamella and primary cell walls of plants, leading to necrosis of plant tissue. In addition, the hydrolysis products of plant cell wall components serve as a food source for bacteria [54]. The isolates we obtained possessed a number of hydrolytic enzymes, pectinases, cellulases, and proteases. Thus, representatives of the genus *Pseudomonas* demonstrated pectinase activity, and some strains also possessed cellulase and protease activities. The presence of these enzymes in pseudomonads has been demonstrated previously. For example, the presence of cellulases and hemicellulases, xylanases, and proteases has been established for representatives of *Pseudomonas fluorescens* and *Pseudomonas syringae* [55]. Pectate lyase has been shown to be involved in the manifestation of virulence in the phytopathogenic bacterium *Pseudomonas viridiflava* [56].

The isolated *Klebsiella* sp. strain PhalM5 exhibited pectolytic and cellulase activities, but no protease activity was detected. Representatives of the genus *Klebsiella* possess enzymes that degrade plant cell walls, in particular, pectate lyases [57], cellulases [58], polygalacturonases, and proteases [59].

Strain *Paracidovorax* sp. PL15 was found to possess pectolytic and cellulolytic activities. Members of the genus *Paracidovorax* are typical plant pathogens characterized by the presence of PCWDEs, including pectate lyases and proteases [60,61].

Strains of the genera *Bacillus* and *Paenibacillus* demonstrated pectolytic and cellulolytic enzyme activities, and some strains also exhibited protease activity. The presence of PCWDEs (pectate lyases, polygalacturonases, cellulases, pectin methyl esterases, and proteases) is characteristic of members of these genera and has been demonstrated in a number of studies [62,63].

The bacterium *Microbacterium* sp. Rs8 demonstrated only pectolytic activity, although some publications have indicated the ability of some members of the genus to synthesize cellulase [64]. We demonstrated the ability of bacteria of the genus *Psychrobacillus* to synthesize pectolytic and cellulolytic enzymes. Although little data are available on the interactions of representatives of this genus with plants, they have been found to contain β-glucosidase enzymes involved in the breakdown of cellulose [65].

Studies have shown that isolates belonging to the genera *Bacillus*, *Paenibacillus*, and *Pseudomonas* synthesize lipase, an enzyme of the carboxyl esterase class that breaks down long-chain glycerides into fatty acids and glycerol. The ability to produce this enzyme is characteristic of these microorganisms; however, little is known about the role of lipase in plant infection by phytopathogenic bacteria. Lipases can be involved in the destruction of plant cuticle layers consisting of waxes and lipid polymers [66]. Lipases have been shown to play a significant role in the development of phytopathogenesis [67].

Biofilm formation and motility are also considered important factors in the virulence of plant pathogens [68,69]. Biofilms promote plant colonization, increase bacterial resistance to unfavorable environmental conditions, and impede the flow of nutrients into plants by clogging vascular tissues, thus playing a significant role in the development of phytopathogenesis [68].

In our study, isolated strains of the *Pseudomonas*, *Bacillus*, and *Paenibacillus* genera demonstrated the ability to form biofilms. The most pronounced activity was observed in the strains *Pseudomonas* sp. PL26, *Pseudomonas* sp. PL27, *Pseudomonas* sp. PR20, *Bacillus* sp. Rs9, *Bacillus* sp. Zeph1, and *Bacillus* sp. Zeph3, while representatives of the genus *Paenibacillus* formed predominantly weak biofilms. Representatives of the *Pseudomonas aeruginosa* and *Paenibacillus polymyxa* species were shown to form biofilms on the trichomes of *Arabidopsis thaliana*, which contributed to more efficient bacterial colonization of plants [68,70]. The formation of biofilms by *Pseudomonas syringae* pv. *theae* on the surface of tea leaves increased the pathogen’s resistance to unfavorable environmental factors and antimicrobial agents [71].

Motility is one of the main factors facilitating host colonization and, therefore, acts as a virulence factor in phytopathogenic microorganisms. It helps bacterial cells avoid exposure to adverse factors, including toxic compounds and antimicrobials. Motility facilitates spread from infected plants to healthy ones and also participates in the formation of biofilms [72]. Swimming is a type of motility of individual bacterial cells in a liquid medium, while swarming is movement along semi-solid substrates, carried out by a group of cells in a coordinated manner [73]. Evaluation of swimming and swarming motility of pectolytic strains showed that all bacterial strains, except *Klebsiella* sp. PhalM5, exhibited swimming activity. However, swarming was characteristic only of some bacteria of the genera *Bacillus*, *Paenibacillus*, and *Pseudomonas*. The absence of flagellar motility for *Klebsiella* was shown by the authors who described this species of bacteria [74]. For representatives of the genera *Paenibacillus*, *Bacillus*, *Pseudomonas*, *Microbacterium*, and *Psychrobacillus*, according to studies, the presence of both types of flagellar motility is characteristic [73,75,76].

Biofilm formation and swarming processes are collective and depend on bacterial cell–cell communication. They are mediated by the quorum-sensing mechanism [73]. Our study demonstrated that *Bacillus* sp. Rs9, *Bacillus* sp. Zeph1, *Bacillus* sp. Zeph3, and *Pseudomonas* sp. PL27, which form strong biofilms, exhibited swarming motility. Strains PL11, PL17, PL18, and PL19, belonging to the genus *Paenibacillus*, also exhibited swarming motility; however, these strains formed weak biofilms with an OD of less than 0.12. *Pseudomonas* sp. PL26 and *Pseudomonas* sp. PR20, despite producing well-defined biofilms, did not exhibit swarming motility. Thus, a relationship between collective motility and biofilm formation was not demonstrated for all strains. No direct relationship between biofilm formation and swarming ability was observed for the remaining bacteria.

Primary screening of pectolytic strains for phytotoxicity was carried out using potato microplants under in vitro conditions. It was found that 20 strains belonging to the genera *Paenibacillus*, *Psychrobacillus*, *Bacillus*, *Pseudomonas*, and *Klebsiella* significantly reduced the values of morphometric parameters: root and shoot length and weight. Moreover, six pectolytic strains that inhibited the growth of potato microplants (strains *Pseudomonas* sp. PL4, *Pseudomonas* sp. PL26, *Pseudomonas* sp. PL27, *Pseudomonas* sp. PR20, *Bacillus* sp. Zeph1, and *Paenibacillus* sp. PL2) possessed the ability to form biofilms, while strains *Bacillus* sp. Zeph1 and *Pseudomonas* sp. PL27 additionally demonstrated swarming motility. However, 14 strains that inhibited the development of potato microplants did not exhibit the ability to form biofilms or swarm. Thus, we did not identify a clear relationship between phytotoxicity towards potato microplants, motility, and biofilm formation. However, it should be noted that strains that did not demonstrate growth inhibition in potato microplants could also potentially be involved in the development of the disease in orchids. Plant species differences may have prevented the bacteria from exhibiting their phytotoxic properties. Therefore, next, all pectolytic strains isolated from orchids exhibiting bacterial disease symptoms should be tested for virulence against *Phalaenopsis*.

An in planta experiment on *Phalaenopsis* Blume plants demonstrated that representatives of the genera *Bacillus*, *Paenibacillus*, *Microbacterium*, and *Psychrobacillus* cause severe waterlogging of plant tissue at the site of leaf inoculation, followed by the development of necrosis. Bacteria of the genera *Microbacterium* and *Psychrobacillus*, most representatives of the genus *Paenibacillus* (except strains PL2, PL17, and PL18), and the strain *Bacillus* sp. M3-3, which cause the formation of pronounced necrotic spots, did not demonstrate the formation of strong or moderately pronounced biofilms and did not possess swarming motility. Injection of phalaenopsis with a suspension of strains of the genera *Klebsiella* and *Paracidovorax* also leads to the appearance of necrotic spots on the upper and lower surfaces of the leaf, with deepening in the form of “holes” caused by the destruction of the parenchyma. The strain *Klebsiella* sp. PhalM5 formed a weak biofilm and was non-motile, while *Paracidovorax* sp. PL15 produced a moderately formed biofilm and exhibited swimming-type motility.

Strains *Bacillus* sp. Rs9, *Paenibacillus* sp. L2, *Pseudomonas* sp. PL4, *Pseudomonas* sp. PL26, and *Pseudomonas* sp. PL27 did not cause visible changes in plant tissue, despite their pronounced ability to form biofilms. Thus, a link between necrosis and social behavior of bacteria was observed only for strains *Paenibacillus* sp. PL2, *Bacillus* Zeph1, and *Bacillus* Zeph3.

Based on the above, it can be concluded that there is no direct relationship between biofilm formation, motility, and the formation of necrotic spots on orchid leaves. Motility and biofilms are necessary for successful colonization of the plant and do not in themselves ensure bacterial virulence [77]. Probably, the presence of enzymes that destroy the plant cell wall is a more significant factor in the formation of leaf necrosis or the suppression of microplant growth. Thus, it was shown that all strains that affect phalaenopsis in planta possessed pectolytic and cellulase activity, or only pectolytic activity (such as strains *Pseudomonas* sp. PR20, *Pseudomonas* sp. PhalM4, and *Microbacterium* Rs8). However, the presence of enzymes does not always determine the presence of necrotic potential in bacteria. The strain *Bacillus* sp. Rs9, which has pronounced cellulase and pectinase activity, high ability to form biofilms and motility, did not have a negative effect on potato and phalaenopsis plants under both in vitro and in planta conditions. Thus, among the pectolytic strains studied, representatives of the genera *Paenibacillus*, *Bacillus*, *Paracidovorax*, *Psychrobacillus*, and *Microbacterium* had the most pronounced effect on *Phalaenopsis* plants. The effect was less pronounced with the strain *Pseudomonas* sp. PhalM4.

The effects of the studied pectolytic strains on potato and *Phalaenopsis* plants varied. Strains that most actively inhibited the growth of potato microplants (*Klebsiella* sp. PhalM5, *Pseudomonas* sp. PL27, *Paenibacillus* sp. PL5, PL6, and PL23) either had no effect on orchid leaves when injected or were significantly weaker than other strains. Meanwhile, strains that promoted the growth of potato microplants (*Microbacterium* sp. Rs8, *Paenibacillus* sp. M3-1, and *Paenibacillus* sp. M3-6) showed the greatest damage to orchid leaves. However, strains such as *Paenibacillus* sp. PL17 and PL18 were active against both potato microplants and orchid leaves. This may indicate a lack of strict host plant species specificity among *Paenibacillus* species. These results suggest that using microplants as a model system for studying potential orchid phytopathogens has limitations due to the specific phytoimmune responses and susceptibility to bacteria in potato and *Phalaenopsis*.

Therefore, we can conclude that the bacteria we studied, belonging to the genera *Paenibacillus*, *Bacillus*, *Microbacterium*, *Psychrobacillus*, and *Klebsiella*, are capable of forming biofilms and synthesizing hydrolytic enzymes, which determines their endophytic lifestyle. It can be hypothesized that under changing plant growing conditions, stress, decreased phytoimmunity, these microorganisms may exhibit toxicity to plants and cause plant tissue necrosis. However, further research is needed to definitively prove that these isolates can act as causative agents of soft rot in orchids under greenhouse conditions.

## 5. Conclusions

Herein we report that bacteria of the genera *Paenibacillus*, *Bacillus*, *Microbacterium*, *Psychrobacillus*, and *Klebsiella*, previously described as endophytes, are associated with bacterial soft rot in orchids. These strains were isolated from *Phalaenopsis* plants with soft rot symptoms and possessed pectolytic activity. Taxonomic identification of the isolates was based on nucleotide sequence analysis of the 16S rRNA gene. The strains exhibited phytotoxicity toward potato microplants and, when injected into orchid leaves, caused signs of plant tissue maceration, suggesting the potential phytopathogenicity of these bacteria. These data confirm the potential phytotoxicity of the isolated bacterial strains, but more detailed studies are needed to definitively establish these isolates as soft rot pathogens under greenhouse conditions. Presumably, the diagnostic and preventative system for bacterial diseases in *Phalaenopsis* spp. plants requires further development to identify new potential groups of phytopathogens. Further detailed studies of the bacteria we isolated are also needed in the future to identify factors influencing the development of disease symptoms in plants.

## Figures and Tables

**Table 1:** Enzymatic activity of bacterial pectolytic strains isolated from *Phalaenopsis* spp.

Strain	Cellulase, EI *	Amylase, EI	Sucrase	Proteinase, EI	Lipase	Oxidase	Catalase
*Bacillus* sp. M3-3	1.67	2.17	−	1.83	+	+	+
*Bacillus* sp. Rs9	4.25	0	+	0	−	+	+
*Bacillus* sp. Zeph1	3.6	0	+	0	+	+	+
*Bacillus* sp. Zeph3	3.2	0	+	0	+	+	+
*Klebsiella* sp. PhalM5	1.33	2.33	+	0	−	+	+
*Microbacterium* sp. Rs8	0	0	−	0	−	+	+
*Paenibacillus* sp. L2	1.67	1.67	+	0	+	−	−
*Paenibacillus* sp. M3-1	2.75	1.33	−	1.33	+	+	+
*Paenibacillus* sp. M3-6	2.0	0	+	0	+	+	+
*Paenibacillus* sp. PL2	2.89	2.36	+	1.6	+	+	+
*Paenibacillus* sp. PL5	4.0	2.18	+	1.2	+	+	+
*Paenibacillus* sp. PL6	3.78	2.67	+	1.4	+	+	+
*Paenibacillus* sp. PL11	3.86	1.86	+	1.2	+	+	+
*Paenibacillus* sp. PL17	3.5	3.00	+	1.25	+	+	+
*Paenibacillus* sp. PL18	3.33	2.00	+	1.2	+	+	+
*Paenibacillus* sp. PL19	6.25	1.56	+	1.33	+	+	+
*Paenibacillus* sp. PL23	4.33	1.27	+	1.2	+	+	+
*Paenibacillus* sp. PR10	3.5	3.2	+	0	+	+	+
*Paenibacillus* sp. PR15	2.8	1.7	+	0	+	+	+
*Paenibacillus* sp. PR16	3.5	1.875	+	0	+	+	+
*Paracidovorax* sp. PL15	2.6	0	−	0	+	+	+
*Pseudomonas* sp. PhalM4	0	0	−	0	−	−	+
*Pseudomonas* sp. PhalM12	2.4	2.3	+	0	+	+	+
*Pseudomonas* sp. PL4	0	0	−	2.33	−	+	+
*Pseudomonas* sp. PL26	1.33	0	+	1.83	+	+	+
*Pseudomonas* sp. PL27	1.5	0	+	2.0	+	+	+
*Pseudomonas* sp. PR20	0	0	−	2.0	+	+	+
*Psychrobacillus* sp. N2-3	2.0	1.33	+	0	−	−	+
*Psychrobacillus* sp. N2-6	1.5	1.33	+	0	−	−	+

* The enzyme activity index (EI) was calculated using the following formula: EI = (colony diameter + clear zone)/colony diameter. “+” and “−” indicate presence and absence of enzyme activities in the tests for sucrase, lipase, oxidase, and catalase.

**Table 2:** The ability of bacterial strains to form biofilms, swimming, and swarming.

Strain	Biofilm-Forming Ability, OD_595_	Swimming, mm^2^	Swarming, mm^2^
*Pseudomonas aeruginosa* PAO1	0.198 ± 0.039	>2000	254
*Bacillus* sp. M3-3	0.109 ± 0.006	>2000	13
*Bacillus* sp. Rs9	1.451 ± 0.073	1661	>2000
*Bacillus* sp. Zeph1	0.703 ± 0.068	1134	>2000
*Bacillus* sp. Zeph3	0.846 ± 0.075	1256	>2000
*Klebsiella* sp. PhalM5	0.100 ± 0.011	28	13
*Microbacterium* sp. Rs8	0.093 ± 0.009	>2000	28
*Paenibacillus* sp. L2	0.095 ± 0.010	>2000	254
*Paenibacillus* sp. M3-1	0.071 ± 0.014	>2000	28
*Paenibacillus* sp. M3-6	0.068 ± 0.005	908	13
*Paenibacillus* sp. PL2	0.352 ± 0.046	380	28
*Paenibacillus* sp. PL5	0.094 ± 0.006	1017	28
*Paenibacillus* sp. PL6	0.103 ± 0.007	>2000	28
*Paenibacillus* sp. PL11	0.108 ± 0.003	908	>2000
*Paenibacillus* sp. PL17	0.111 ± 0.006	804	707
*Paenibacillus* sp. PL18	0.109 ± 0.004	804	>2000
*Paenibacillus* sp. PL19	0.078 ± 0.005	452	1661
*Paenibacillus* sp. PL23	0.109 ± 0.004	314	28
*Paenibacillus* sp. PR10	0.081 ± 0.004	1256	28
*Paenibacillus* sp. PR15	0.067 ± 0.002	707	12
*Paenibacillus* sp. PR16	0.074 ± 0.003	615	28
*Paracidovorax* sp. PL15	0.134 ± 0.015	>2000	28
*Pseudomonas* sp. PhalM4	0.321 ± 0.017	>2000	12
*Pseudomonas* sp. PhalM12	0.076 ± 0.008	>2000	12
*Pseudomonas* sp. PL4	0.243 ± 0.013	1385	12
*Pseudomonas* sp. PL26	0.870 ± 0.078	615	28
*Pseudomonas* sp. PL27	0.634 ± 0.006	908	113
*Pseudomonas* sp. PR20	0.604 ± 0.040	1134	28
*Psychrobacillus* sp. N2-3	0.167 ± 0.011	1809	3
*Psychrobacillus* sp. N2-6	0.134 ± 0.012	1256	28

**Table 3:** Morphometric parameters of 30-day-old potato (cultivar Nevsky) microplants without inoculation (control) and inoculated with bacterial pectolytic strains (10^6^ cells/mL).

Strain	Shoot Length, mm	Root Length, mm	Fresh Shoot Weight, mg	Fresh Root Weight, mg	DI,%
Control	102.6 ± 8.9 ^g^ *	52.8 ± 6.1 ^hijkl^	386 ± 15 ^L^	109.4 ± 15.5 ^i^	0
*Bacillus* sp. M3-3	**83.4 ± 2.6** ^bcdef^ **	44.6 ± 5.2 ^efghij^	**311 ± 19** ^efghijk^	**55.7 ± 9.7** ^efg^	0
*Bacillus* sp. Rs9	**82.7 ± 3.3** ^bcdef^	47.5 ± 3.3 ^fghijk^	374 ± 16 ^kL^	**69.7 ± 5.8** ^fgh^	0
*Bacillus* sp. Zeph1	85.7 ± 3.9 ^bcdefg^	60.7 ± 4.9 ^lmn^	**296 ± 21** ^defghij^	**28.1 ± 2.9** ^abcde^	0
*Bacillus* sp. Zeph3	**84.0 ± 4.0** ^bcdef^	*67.1 ± 8.5* ^mno^ ***	**312 ± 18** ^efghijk^	102.5 ± 12.8 ^i^	4.17
*Klebsiella* sp. PhalM5	**49.3 ± 3.8** ^a^	**28.3 ± 5.4** ^abcd^	**159 ± 16** ^a^	**5.2 ± 0.7** ^a^	77.8
*Microbacterium* sp. Rs8	94.7 ± 5.3 ^defg^	*69.9 ± 8.7* ^no^	318 ± 16 ^fghijkl^	82.9 ± 5.8 ^ghi^	4.17
*Paenibacillus* sp. L2	**83.9 ± 6.2** ^bcdef^	62.6 ± 9.1 ^lmn^	**292 ± 24** ^defghi^	100.8 ± 20.9 ^hi^	0
*Paenibacillus* sp. M3-1	88.1 ± 4.2 ^bcdefg^	*72.3 ± 3.9* ^no^	320 ± 16 ^fghijkl^	83.0 ± 8.6 ^ghi^	6.94
*Paenibacillus* sp. M3-6	93.6 ± 7.2 ^defg^	*68.2 ± 5.7* ^mno^	352 ± 47 ^ijkl^	*157.7 ± 20.9* ^j^	0
*Paenibacillus* sp. PL2	92.0 ± 5.2 ^cdefg^	44.9 ± 6.7 ^efghij^	**234 ± 18** ^bcd^	**8.7 ± 1.8** ^ab^	23.6
*Paenibacillus* sp. PL5	**84.3 ± 6.1 ^bcdef^**	**24.7 ± 4.2 ^ab^**	**218 ± 16** ^abc^	**5.2 ± 1.1** ^a^	8.8
*Paenibacillus* sp. PL6	**74.9 ± 7.1 ^bc^**	**20.8 ± 3.4 ^a^**	**216 ± 17** ^ab^	**13.6 ± 2.3** ^abc^	8.8
*Paenibacillus* sp. PL11	87.0 ± 4.9 ^bcdefg^	**26.1 ± 2.2 ^abc^**	**244 ± 19** ^bcde^	**8.21 ± 1.6** ^ab^	20
*Paenibacillus* sp. PL17	**85.3 ± 5.8 ^bcdef^**	**25.4 ± 3.0 ^abc^**	**248 ± 10** ^bcde^	**6.9 ± 1.1** ^a^	8.8
*Paenibacillus* sp. PL18	**79.6 ± 7.3 ^bcd^**	**27.5 ± 3.8 ^abcd^**	**228 ± 13** ^abcd^	**29.3 ± 2.3** ^abcde^	5.5
*Paenibacillus* sp. PL19	92.2 ± 6.0 ^defg^	**35.3 ± 3.0 ^bcdef^**	**295 ± 25** ^defghij^	**14.2 ± 2.2** ^abc^	16.7
*Paenibacillus* sp. PL23	88.8 ± 5.1 ^cdefg^	**33.0 ± 7.7 ^abcde^**	**273 ± 17** ^bcdefgh^	**12.5 ± 2.9** ^abc^	33.3
*Paenibacillus* sp. PR10	86.6 ± 5.5 ^bcdefg^	60.4 ± 4.8 ^klmn^	**258 ± 21** ^bcdef^	**28.9 ± 6.2** ^abcde^	4.17
*Paenibacillus* sp. PR15	96.9 ± 5.6 ^efg^	50.7 ± 6.0 ^ghijkl^	340 ± 12 ^hijkl^	**32.4 ± 5.2** ^abcde^	16.7
*Paenibacillus* sp. PR16	93.0 ± 5.6 ^defg^	50.5 ± 6.0 ^ghijkl^	**237 ± 9** ^bcd^	**31.4 ± 3.3** ^abcde^	11.1
*Paracidovorax* sp. PL15	96.9 ± 5.5 ^efg^	43.0 ± 5.0 ^efghi^	340 ± 13 ^hijkl^	**29.0 ± 2.8** ^abcde^	0
*Pseudomonas* sp. PhalM4	97.6 ± 3.5 ^efg^	*76.2 ± 5.4* ^o^	318 ± 20 ^fghijkl^	**70.0 ± 24.**5 ^fgh^	0
*Pseudomonas* sp. PhalM12	**81.7 ± 4.9 ^bcde^**	44.6 ± 3.6 ^efghij^	**289 ± 24** ^defghi^	**51.9 ± 16.2** ^defg^	0
*Pseudomonas* sp. PL4	**71.2 ± 4.8 ^b^**	40.2 ± 5.7 ^defgh^	**287 ± 22** ^cdefghi^	**54.9 ± 9.4** ^efg^	5.5
*Pseudomonas* sp. PL26	**78.1 ± 8.3 ^bcd^**	**37.8 ± 4.3** ^cdefg^	**229 ± 26** ^bcd^	**18.0 ± 5.8** ^abc^	5.5
*Pseudomonas* sp. PL27	**85.3 ± 12.6 ^bcdef^**	53.4 ± 5.7 ^ijkl^	331 ± 35 ^ghijkl^	**42.8 ± 4.6** ^cdef^	0
*Pseudomonas* sp. PR20	87.4 ± 5.9 ^bcdefg^	56.6 ± 5.8 ^jklm^	**267 ± 19** ^bcdefg^	**20.6 ± 4.1** ^abcd^	20.8
*Psychrobacillus* sp. N2-3	99.5 ± 4.8 ^fg^	45.0 ± 4.4 ^efghij^	349 ± 27 ^ijkl^	**39.0 ± 8.6** ^bcdef^	0
*Psychrobacillus* sp. N2-6	93.6 ± 5.2 ^defg^	50.1 ± 3.5 ^ghijkl^	363 ± 15 ^jkl^	**64.3 ± 7.9** ^fg^	0

* Data are presented as the mean  ±  confidence interval for a 95% significance level. The letters a, b, c, etc., refer to the sets of data that differed significantly when ANOVA yielded a significant result. ** Values shown in bold are those that are significantly lower than the control value according to the Tukey test (*p* ≤ 0.05). *** Values shown in italics and underlined are those that are significantly higher than the control value according to the Tukey test (*p* ≤ 0.05).

**Table 4:** The symptoms on *Phalaenopsis* sp. leaves in planta inoculated with bacterial pectolytic strains (10^6^ cells/mL).

Strain	Symptoms	Phytotoxicity in planta
Sterile water (control)	No visible changes	− *
*Bacillus* sp. M3-3	A dark green (underside of the leaf) or light brown with a yellow border (upper side of the leaf) wet spot with a diameter of about 3.5 cm	++++
*Bacillus* sp. Rs9	No visible changes	−
*Bacillus* sp. Zeph1	A yellow spot with a diameter of more than 0.7 cm on the upper side of the leaf, individual dark brown dots on the lower side of the leaf	++
*Bacillus* sp. Zeph3	A dark green, water-soaked spot about 2.5 cm in diameter on the upper and lower sides of the leaf	++++
*Klebsiella* sp. PhalM5	A brown pit-shaped spot about 0.5 cm in diameter with pronounced tissue necrosis	+
*Microbacterium* sp. Rs8	A light-brown, water-soaked spot (2.5 cm) with a yellow border and a pronounced necrotic zone on the upper side of the leaf; on the lower side of the leaf there is a light-brown spot with a yellow border	++++
*Paenibacillus* sp. L2	No visible changes	−
*Paenibacillus* sp. M3-1	A dark green spot with an olive border (about 3 cm in diameter) on the upper side of the leaf and dark green, water-soaked spot on the lower side of the leaf	++++
*Paenibacillus* sp. M3-6	A dark green spot with a yellowish border (about 3 cm in diameter) on the upper side of the leaf, and dark green, water-soaked spot on the lower side of the leaf	++++
*Paenibacillus* sp. PL2	Dark green, water-soaked spots about 2 cm in diameter on the upper and lower sides of the leaf	+++
*Paenibacillus* sp. PL5	Yellow-green spots more than 0.7 cm in diameter on the upper and lower sides of the leaf	++
*Paenibacillus* sp. PL6	Yellow-green spots up to 0.5 cm in diameter on the upper and lower sides of the leaf	+
*Paenibacillus* sp. PL11	Spots up to 1.5 cm in diameter with necrotic brown zones in the center and yellow borders on the upper and lower sides of the leaf	++
*Paenibacillus* sp. PL17	A black, water-soaked spot about 3 cm in diameter with a yellow border	+++
*Paenibacillus* sp. PL18	Irregularly shaped spot (approx. 2 cm in diameter) with a dark necrotic zone, a light brown macerated zone, and a yellow border	+++
*Paenibacillus* sp. PL19	Yellow-green spots up to 0.5 cm in diameter on the upper and lower sides of the leaf	+
*Paenibacillus* sp. PL23	Dark green, water-soaked spots about 2 cm in diameter on the upper and lower sides of the leaf with a pronounced zone of brown necrosis	+++
*Paenibacillus* sp. PR10	Dark green, water-soaked spots up to 3 cm in diameter with a yellowish border, with a depression at the injection site	++++
*Paenibacillus* sp. PR15	Minor radial darkening of the upper side of the leaf with slight signs of tissue waterlogging	++
*Paenibacillus* sp. PR16	A 2 cm diameter water-soaked spot on the underside of a leaf	++++
*Paracidovorax* sp. PL15	A yellow spot 1.5 cm in diameter with a distinct depression in the center, without traces of tissue hydration	+++
*Pseudomonas* sp. PhalM4	A light brown spot up to 1 cm in diameter on the upper side of the leaf, a water-soaked olive-colored spot about 2 cm in diameter on the underside of the leaf	+++
*Pseudomonas* sp. PhalM12	A yellow-green spot up to 1.5 cm in diameter on the upper side of the leaf, a water-soaked spot on the underside	++
*Pseudomonas* sp. PL4	No visible changes	−
*Pseudomonas* sp. PL26	No visible changes	−
*Pseudomonas* sp. PL27	No visible changes	−
*Pseudomonas* sp. PR20	A yellow spot with a diameter of 0.5 cm on the upper side of the leaf without traces of tissue waterlogging	+
*Psychrobacillus* sp. N2-3	A slight change in pigmentation of the upper side of the leaf to a yellowish tint, individual dark spots on the lower side of the leaf	++
*Psychrobacillus* sp. N2-6	Dark green, water-soaked spots (up to 2.5 cm in diameter) with a yellow halo on the upper and lower sides of the leaf. A depression forms in the center of the spot on the lower side	++++

− *—no visible changes in plant tissue; +—necrotic spots up to 0.7 cm in diameter or a change in leaf pigmentation at the site of inoculation; ++—necrotic spots up to 1 cm in diameter; +++—water-soaked spots 1–2 cm in diameter; ++++—water-soaked spots more than 2 cm in diameter.

## Data Availability

The sequences of bacterial 16S rRNA genes presented in this study are openly available in the GenBank database at https://www.ncbi.nlm.nih.gov/ (accessed on 13 June 2026), reference numbers PX277000.1; PX277018.1; PX277003.1; PX277004.1; PX277014.1; PX277002.1; PX277029.1; PX277023.1; PX277024.1; PX277006.1; PX277007.1; PX277005.1; PX277001.1; PX277011.1; PX277012.1; PX277010.1; PX277009.1; PX277027.1; PX277028.1; PX277026.1; PX277008.1; PX277022.1; PX277016.1; PX277020.1; PX277017.1; PX277015.1; PX277013.1; PX277019.1; PX277021.1.

## Supplementary Materials

The following supporting information can be downloaded at: https://www.mdpi.com/article/10.3390/plants15121901/s1, Figure S1: 30-day-old microplants of potato cultivar Nevsky that were uninoculated (control, A) and inoculated with 10^6^ cells/mL of *Klebsiella* sp. PhalM5 (B), *Paenibacillus* sp. PL2 (C), and *Paenibacillus* sp. PL23 (D); Figure S2: Leaves of *Phalaenopsis* spp. 5 days after injection by 16 pectolytic strains and sterile water (control) the upper and the lower surfaces of the leaf; Table S1: Sources of isolation of pectolytic bacterial strains from *Phalaenopsis* spp. plants; Table S2: Results of the 16S rRNA test for 29 pectolytic strains isolated from soft-rot *Phalaenopsis* and the bacterial type strains.

## Abbreviations

The following abbreviations are used in this manuscript:

DI	Disease index
EI	Enzyme activity index
LB	Lysogeny broth
OD	Optical density
PCWDE	Plant cell wall-degrading enzymes
