# Supplementary material for: Taxonomy Identification and Phytotoxic Activities of Pectolytic Bacteria Isolated from Diseased Plants of Phalaenopsis Blume (Orchidaceae)
Source: Plants (Basel). 2026 Jun 18;15(12):1901. doi: 10.3390/plants15121901 (PMC13306336; doi:10.3390/plants15121901)
Supplement: Supplementary file 1 [file plants-15-01901-s001.zip › Table S1.pdf]

Table S1. Sources of isolation of pectolytic bacterial strains from *Phalaenopsis* spp. plants

Strain	Batch number	Source of isolation	Isolation data
Bacillus sp. M3-3	3	leaf endosphere	06/2025
Bacillus sp. Rs9	5	root endosphere	06/2025
Bacillus sp. Zeph1	6	leaf episphere	06/2025
Bacillus sp. Zeph3	6	leaf episphere	06/2025
Klebsiella sp. PhalM5	1	leaf episphere	06/2025
Microbacterium sp. Rs8	5	root endosphere	06/2025
Paenibacillus sp. L2	2	leaf endosphere	06/2025
Paenibacillus sp. M3-1	3	leaf endosphere	06/2025
Paenibacillus sp. M3-6	3	leaf endosphere	06/2025
Paenibacillus sp. PL2	7	leaf endosphere	06/2025
Paenibacillus sp. PL5	7	leaf endosphere	06/2025
Paenibacillus sp. PL6	7	leaf endosphere	06/2025
Paenibacillus sp. PL11	7	leaf endosphere	06/2025
Paenibacillus sp. PL17	7	leaf endosphere	06/2025
Paenibacillus sp. PL18	7	leaf endosphere	06/2025
Paenibacillus sp. PL19	7	leaf endosphere	06/2025
Paenibacillus sp. PL23	7	leaf endosphere	06/2025
Paenibacillus sp. PR10	7	root endosphere	06/2025
Paenibacillus sp. PR15	7	root endosphere	06/2025
Paracidovorax sp. PL15	7	leaf endosphere	06/2025
Pseudomonas sp. PhalM4	1	leaf endosphere	06/2025
Pseudomonas sp. PhalM12	1	leaf endosphere	06/2025
Pseudomonas sp. PL4	7	leaf endosphere	06/2025
Pseudomonas sp. PL26	7	leaf endosphere	06/2025
Pseudomonas sp. PL27	7	leaf endosphere	06/2025
Pseudomonas sp. PR20	7	root endosphere	06/2025
Paenibacillus sp. PR16	7	root endosphere	06/2025
Psychrobacillus sp. N2-3	4	leaf endosphere	06/2025
Psychrobacillus sp. N2-6	4	leaf endosphere	06/2025
