# Supplementary material for: Taxonomy Identification and Phytotoxic Activities of Pectolytic Bacteria Isolated from Diseased Plants of Phalaenopsis Blume (Orchidaceae)
Source: Plants (Basel). 2026 Jun 18;15(12):1901. doi: 10.3390/plants15121901 (PMC13306336; doi:10.3390/plants15121901)
Supplement: Supplementary file 1 [file plants-15-01901-s001.zip › Figure S2.pdf]

Bacillus sp. M3-3

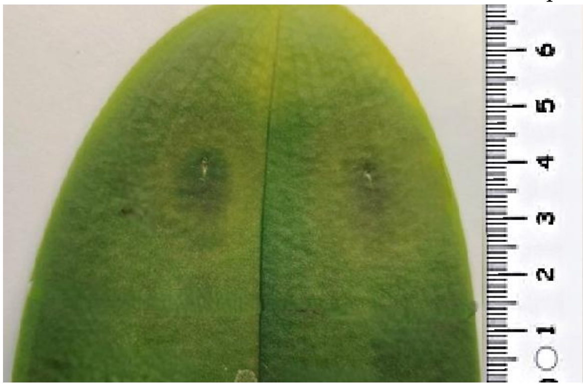
the upper surface of the leaf

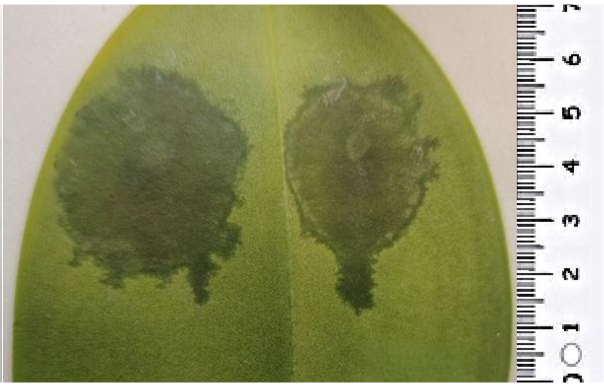
the lower surface of the leaf

Bacillus sp. Zeph 3

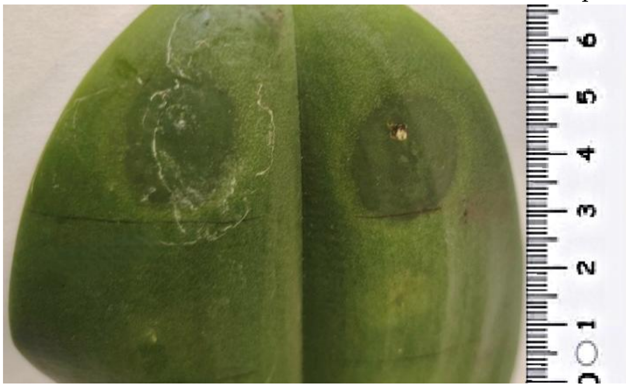
the upper surface of the leaf

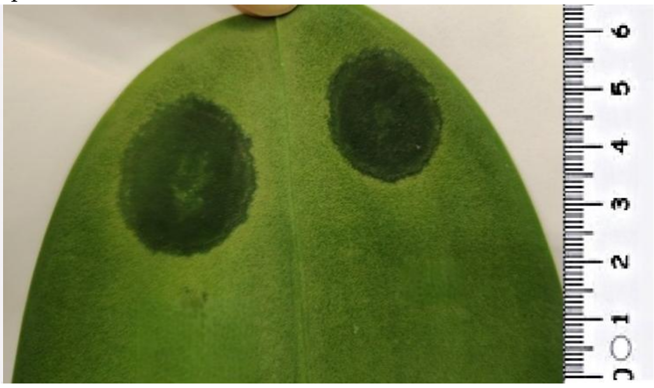
the lower surface of the leaf

Microbacterium sp. Rs8

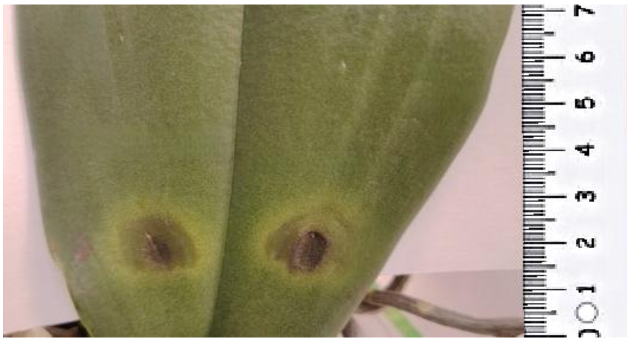
the upper surface of the leaf

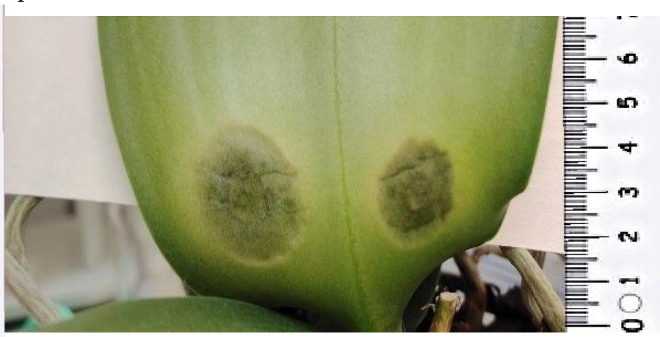
the lower surface of the leaf

Paenibacillus sp. M3-1

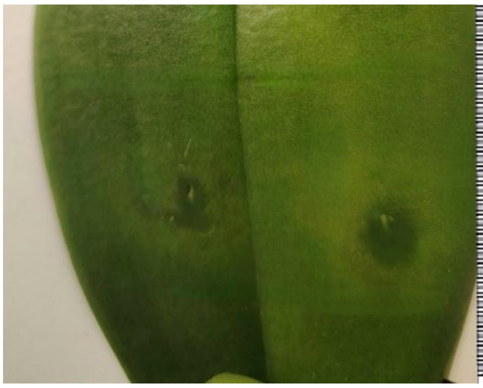
the upper surface of the leaf

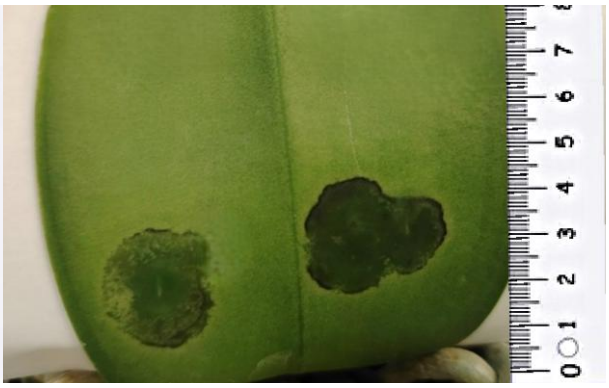
the lower surface of the leaf

Paenibacillus sp. M3-6

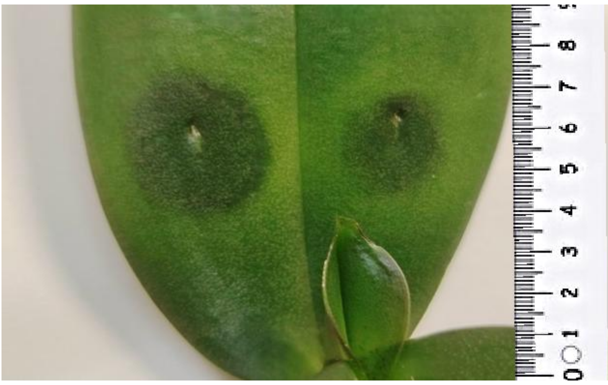

the upper surface of the leaf

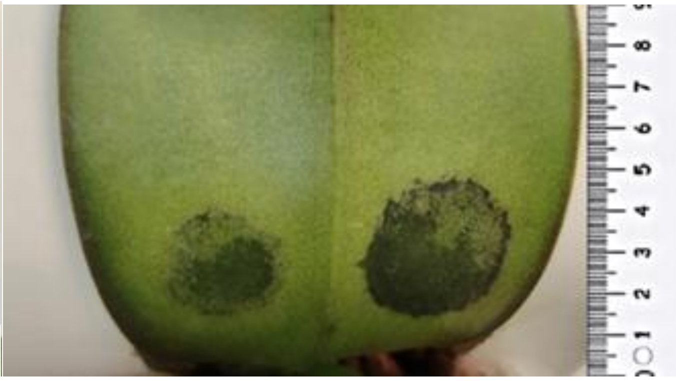

the lower surface of the leaf

Paenibacillus sp. PL2

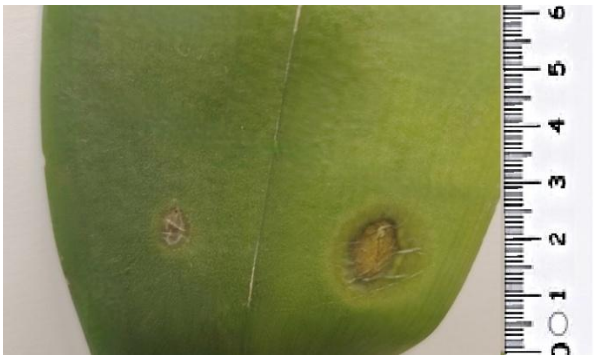

the upper surface of the leaf

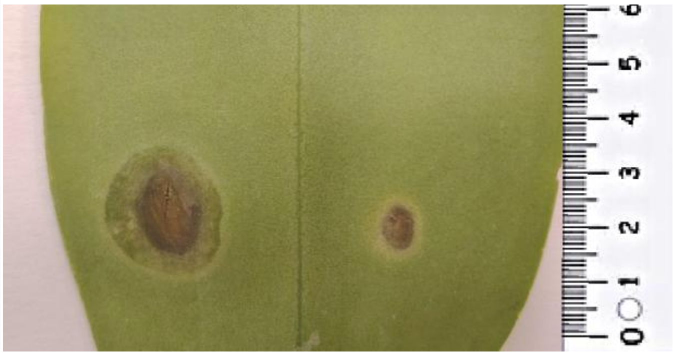

the lower surface of the leaf

Paracidovorax sp. PL15

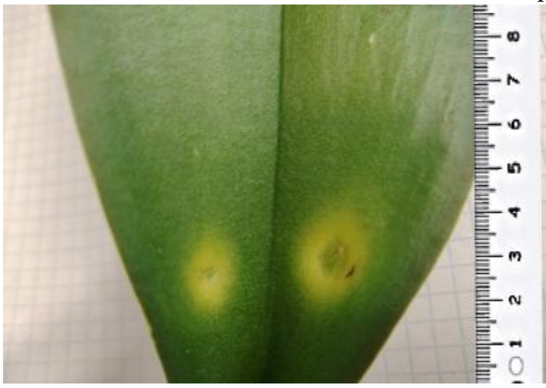

the upper surface of the leaf

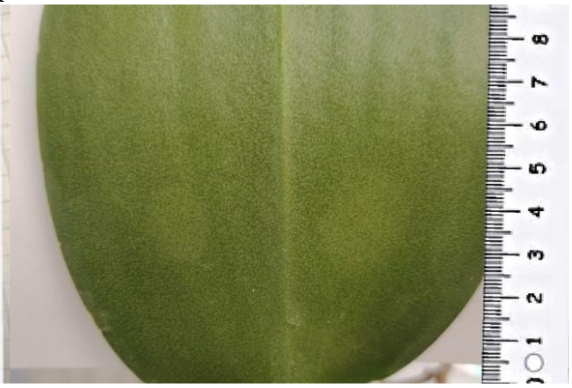

the lower surface of the leaf

Paenibacillus sp. PL17

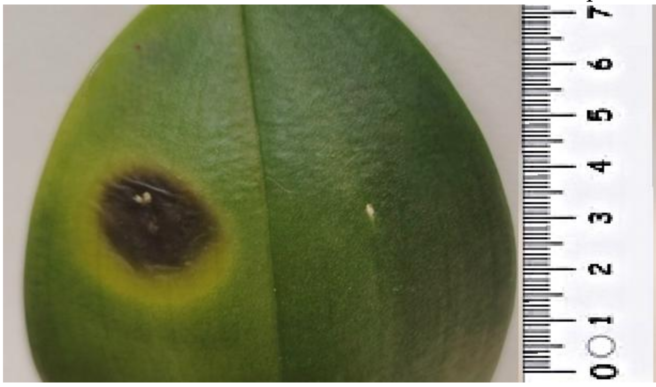

the upper surface of the leaf

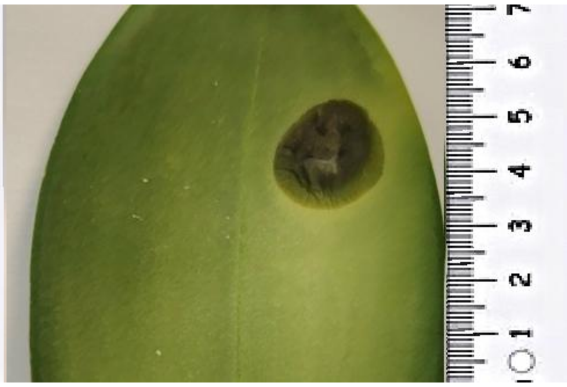

the lower surface of the leaf

Paenibacillus sp. PL18

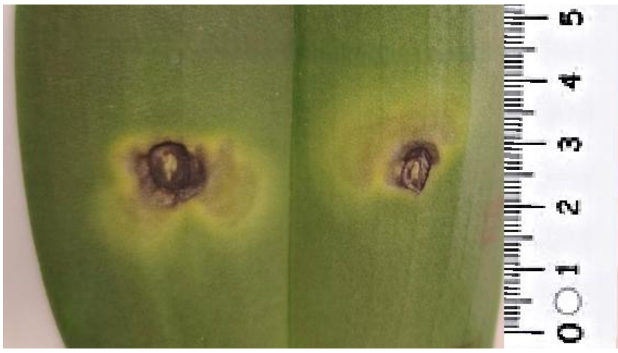

the upper surface of the leaf

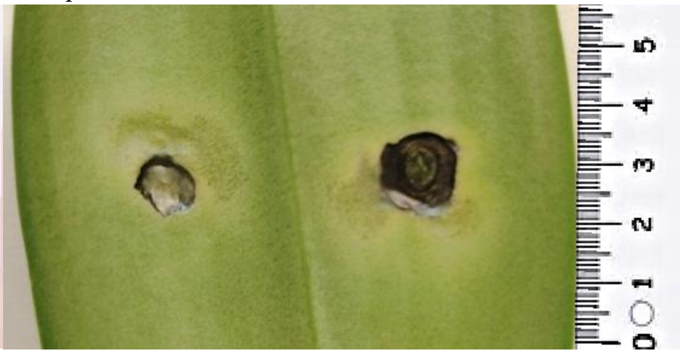

the lower surface of the leaf

Paenibacillus sp. PL23

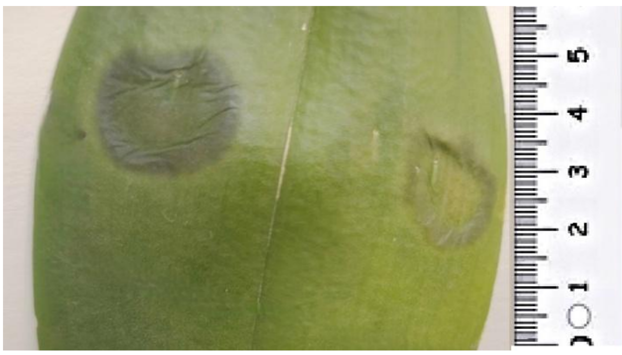

the upper surface of the leaf

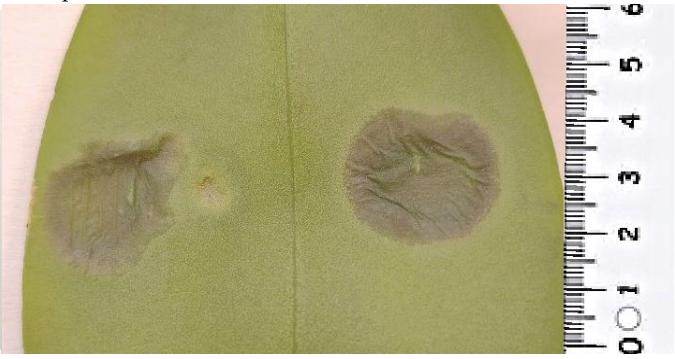

the lower surface of the leaf

Chryseobacterium sp. PR1

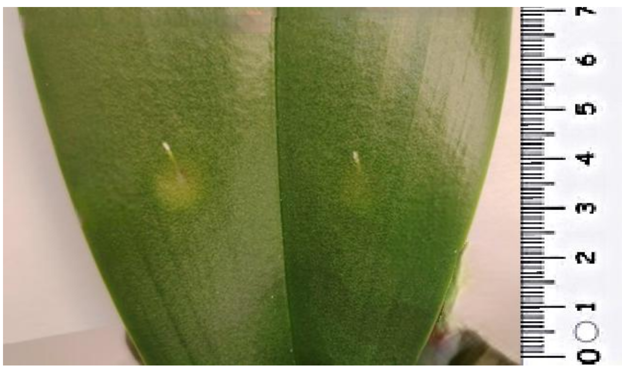

the upper surface of the leaf

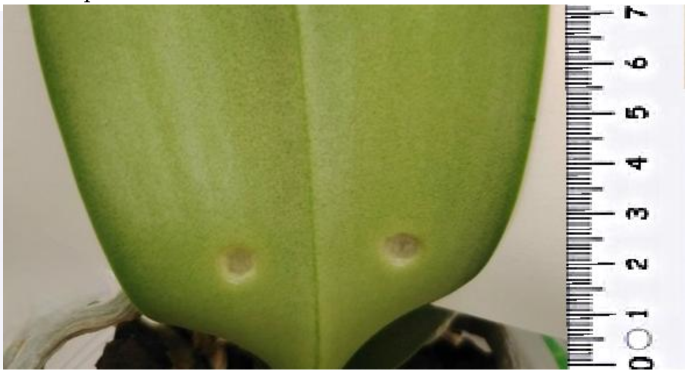

the lower surface of the leaf

Paenibacillus sp. PR10

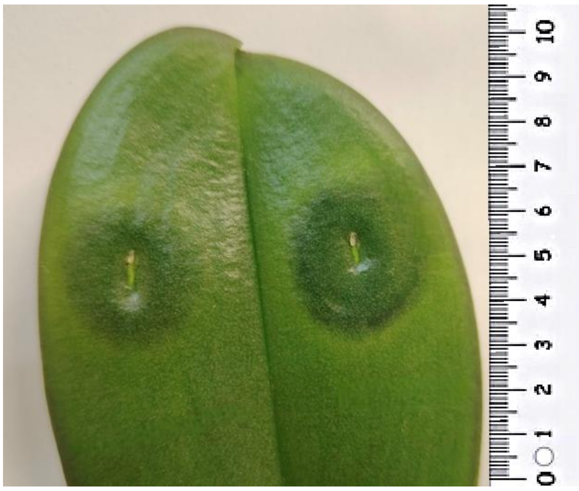

the upper surface of the leaf

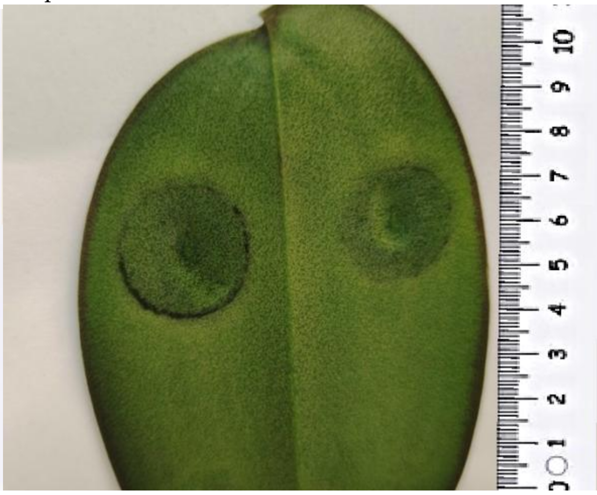

the lower surface of the leaf

Paenibacillus sp. PR16

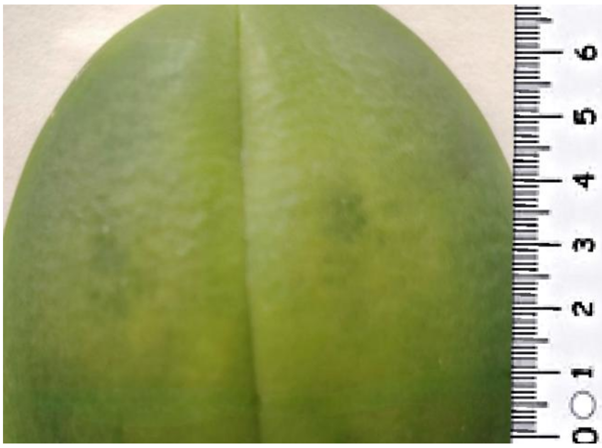

the upper surface of the leaf

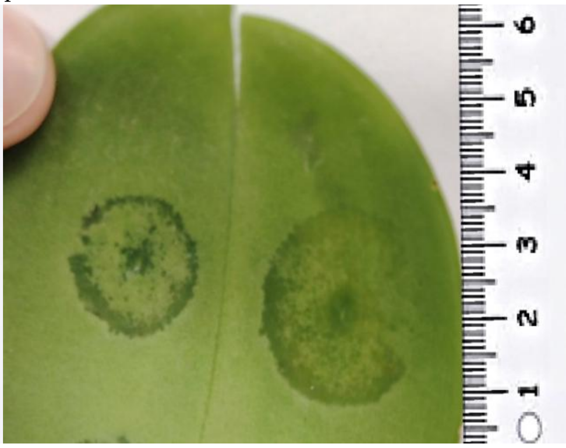

the lower surface of the leaf

Psychrobacillus sp. N2-6

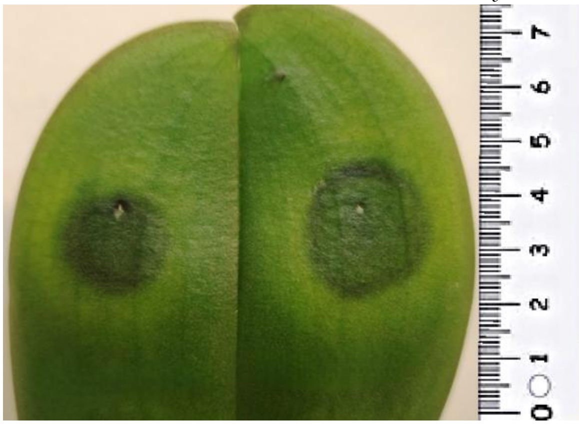

the upper surface of the leaf

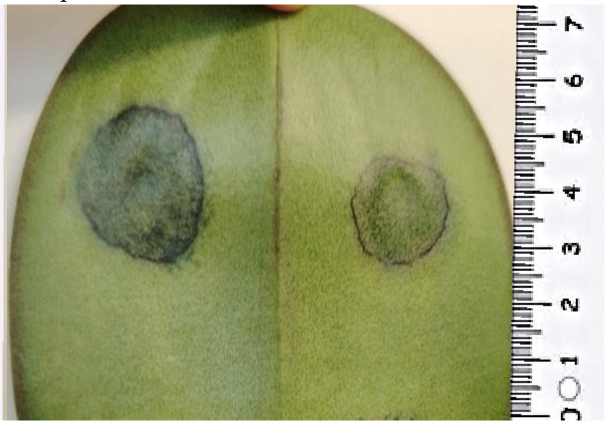

the lower surface of the leaf

Pseudomonas sp. PhalM4

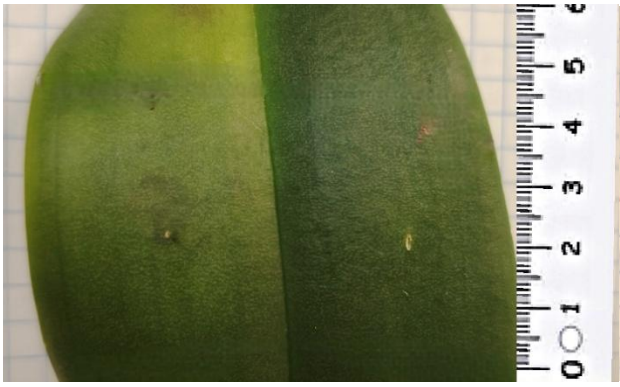

the upper surface of the leaf

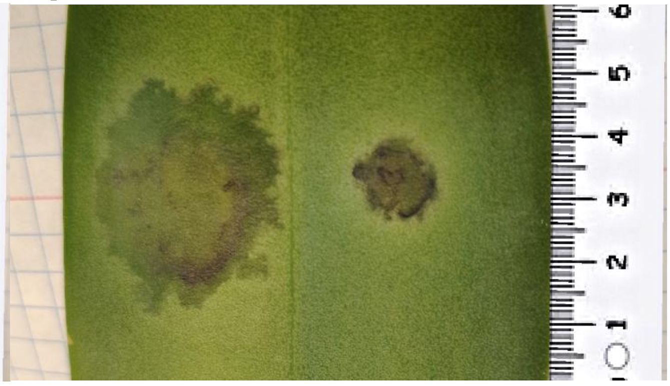

the lower surface of the leaf

Klebsiella sp. PhalM5

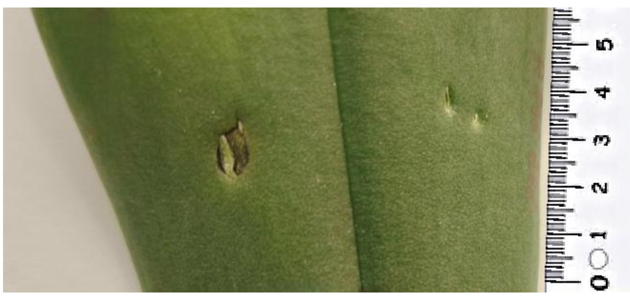

the upper surface of the leaf

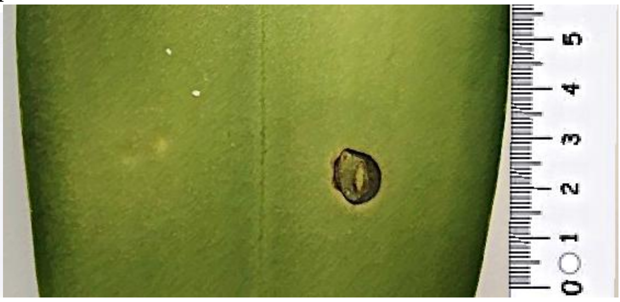

the lower surface of the leaf

Control

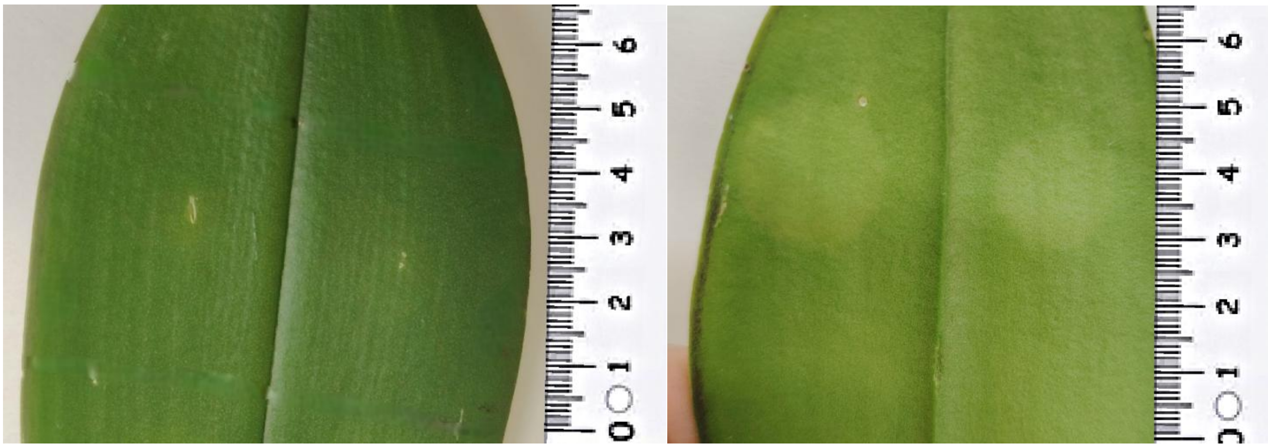

the upper surface of the leaf the lower surface of the leaf

Figure S2. Leaves of *Phalaenopsis* spp. 5 days after injection by 16 pectolytic strains and sterile water (control) the upper and the lower surfaces of the leaf.
