# Supplementary material for: Taxonomy Identification and Phytotoxic Activities of Pectolytic Bacteria Isolated from Diseased Plants of Phalaenopsis Blume (Orchidaceae)
Source: Plants (Basel). 2026 Jun 18;15(12):1901. doi: 10.3390/plants15121901 (PMC13306336; doi:10.3390/plants15121901)
Supplement: Supplementary file 1 [file plants-15-01901-s001.zip › Figure S1.pdf]

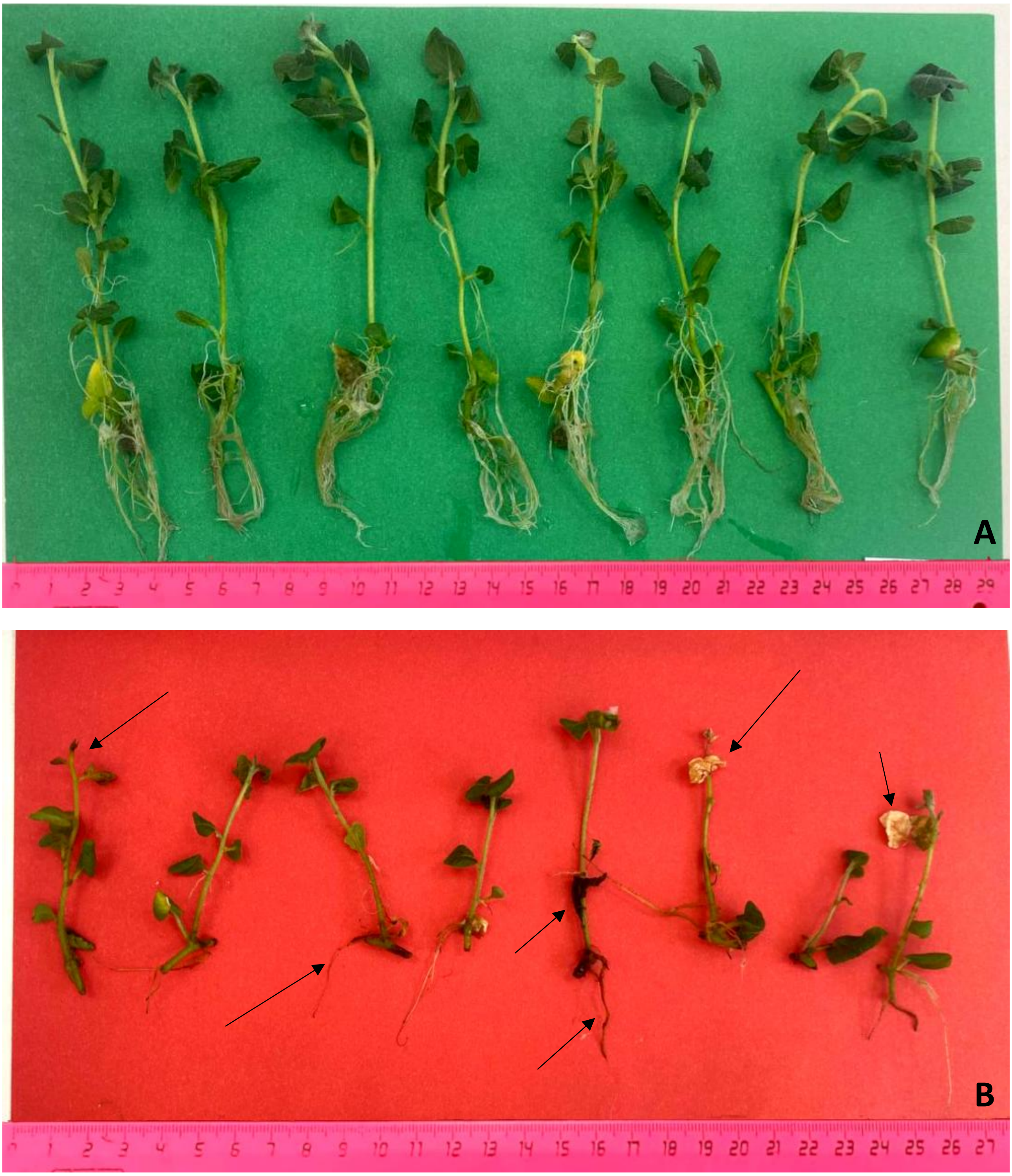
A
B

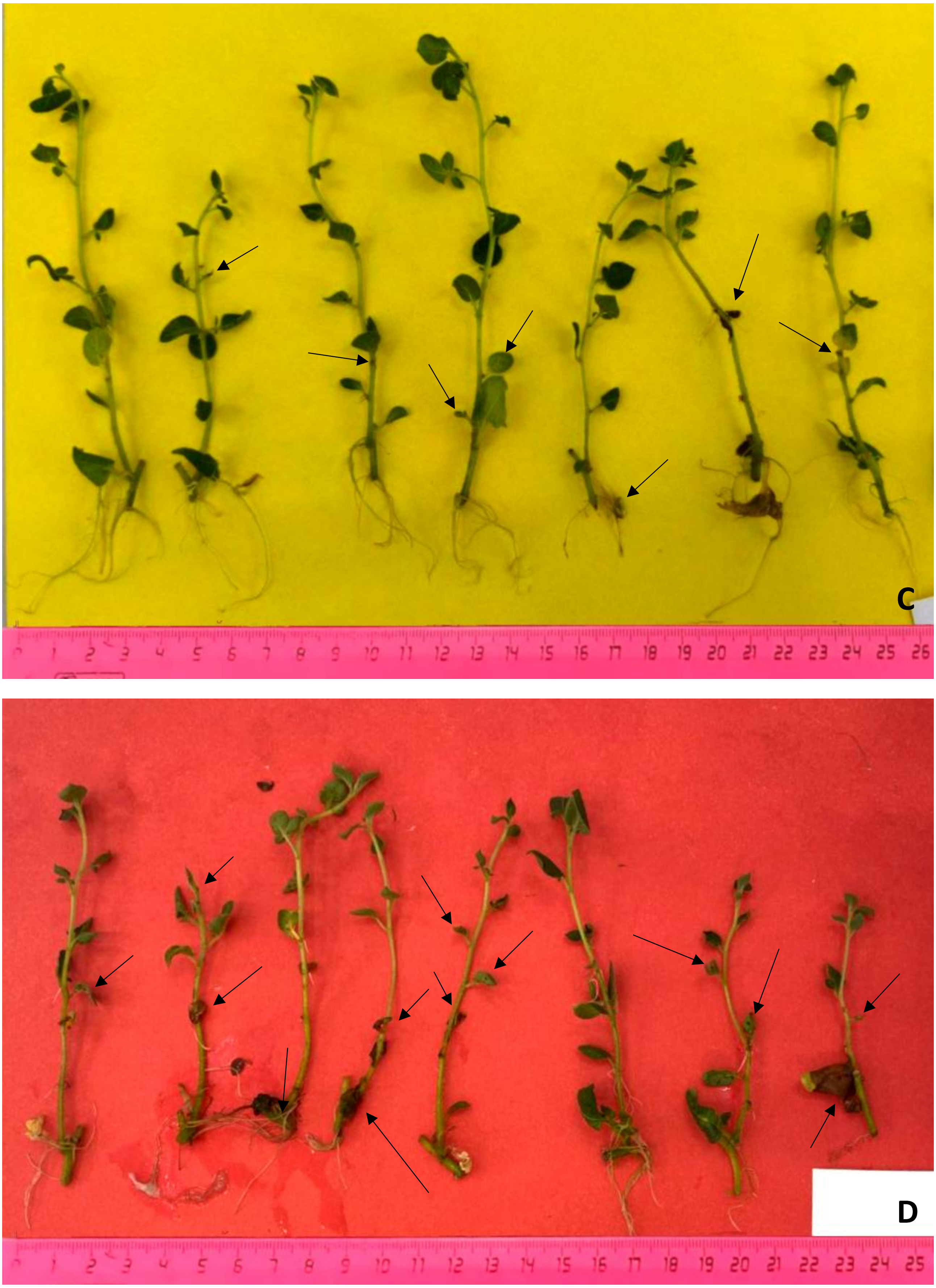

Figure S1. 30-day-old microplants of potato cultivar Nevsky that were uninoculated (control, A) and inoculated with 10^6 cells/mL of *Klebsiella* sp. PhalM5 (B), *Paenibacillus* sp. PL2 (C), and *Paenibacillus* sp. PL23 (D). Arrows indicate spots on leaves and stems, slimming and rotting of roots, and wilting of leaf blades.
